# Higher harmonics and supercontinuum generated from the Kerr response time in different states of matter from a universal electromagnetic model

**DOI:** 10.1038/s41598-023-42579-z

**Published:** 2023-09-19

**Authors:** Robert R. Alfano, Shah Faisal B. Mazhar

**Affiliations:** Institute for Ultrafast Spectroscopy and Lasers-Physics Department, The City College of New York, 160 Convent Avenue, New York, NY 10031 USA

**Keywords:** Optics and photonics, Physics

## Abstract

There is a need for a universal model to describe higher harmonic generation (HHG) in different states of matter. Based on an electromagnetic model (EM), the generation of odd higher harmonic (HHG) and supercontinuum (SC) from intense fs and ps pulses for visible, NIR, and MIR lasers is simulated based on the parameters from experimental observation. HHG and SC depend critically on the different Kerr material response times τ from the ultrafast on the order of 100 as for electronic cloud distortion to fast ~ 10 fs from plasma and molecular redistribution and to the slower picoseconds rotational and vibrational molecular processes. The number of odd HHG generated is shown to depend critically on the fastest Kerr response time on the order of ~ 1 fs from electronic self-phase modulation (ESPM). In this study, different states of matter from noble gas Argon to condensed matter ZnO and LBG are simulated showing the dependence on the Kerr response time to produce HHG for various applications in Physics, Chemistry, Biology, and Engineering. The EM model is universal to produce HHG and SC in different states of matter.

## Introduction

One of the most exciting nonlinear optical effects being pursued is the white light and infrared generation of Supercontinuum (SC) and the odd Higher Harmonic Generation (HHG) in solids, liquids, and gases. It all started 52 years ago with the white light continuum in glasses, crystals, noble gases, and liquids^1–3^. The forerunner of HHG was proposed by Alfano et al. in 1972 that electronic self-phase modulation could generate odd higher harmonics^4^. A year later, Stephen Harris generated vacuum-ultraviolet and soft-X-ray radiation using high-order nonlinear optical polarizabilities^5^. About 30 years ago, experimental observations into the ultraviolet showed the generation of odd harmonics and attosecond pulses from rare gases using extreme ultrafast laser beams^6–11^. The model used to explain the Higher Harmonics Generation (HHG) from semi-classical and time-depended Schrodinger equation (TDSE) derived by Lewenstein et al.^11^ is not suitable to explain the experimental generation of HHG from different states of matter in particular solids (dielectrics and semiconductors) and does not generate even harmonics as observed by most experimentalists^6–11^. Chen et al.^12^ appear to produce theoretical and experimental even and odd harmonics from CO molecules using the Schrodinger equation in the dipole approximation. The resulting HHG from Chen does not clearly show the three salient features of the experimental HHG spectrum. Figure 2 and 3 from Chen’s paper^12^ appear to look more like a noise than the characteristic peaks of HHG with decreasing harmonics followed by plateau region and the cutoff harmonics. Therefore, Chen’s observations using Schrodinger’s equation result in a wide band of frequencies that spans to EUV, and Fig. 2^12^ has harmonics going up and down without any consistent even and odd harmonic pattern. This contrasts with the observation and the work of Reis’ group on a uniaxial crystal of MoS_2_ (see Supplementary Fig. S12; Supplementary Ref. S10).

Corkum and Lewenstein used a semi-classical three-step and a quantum–mechanical model of the Fermi golden rule with Schrodinger dipole E.r approximation for the outer electrons^6, 11^. The cutoff energy of HHG was related to IP + 3UP, ionization (IP), and potential (UP) energy. This energy UP can be regarded as the electron’s ponderomotive energy (also called “quiver energy”) where an oscillating electric field pushes the electron back and forth in a “Quivering” motion. If the electric field pushes hard enough, and the frequency is low enough, then the electron winds up with a lot of kinetic energy (on average) to remove electrons for a single electron model and return to parent ion. This 3-step model has been explained as follows: ionization of the electron, the high-intensity laser field (10^14^ to 10^16^ W/cm^2^); the electron tunnels through the electric potential barrier; acceleration of the free electron, the free electron generated by tunneling with a zero initial velocity is accelerated away from the parent ion by the driven field; then finally, the electron is driven back to and returns to the parent ion. This 3-step model is rather extreme to produce HHG and cannot explain HHG in solids and different states of matter. Moreover, the 3-step process lacks the basic physical insight to explain the production of HHG from new materials such as solids.

Reis’ group and colleagues have been conducting experiments on HHG in solids such as bulk crystals: MgO and ZnO; and 2D layered materials: MoS_2_ and WS_2_^13–17^. Reis pointed out the lack of proper theoretical interpretation of HHG in solid and the underlying mechanism behind it. Furthermore, “the underlying mechanism for HHG is still under debate, and a unified predictive theory that captures the diversity of solids remains elusive”^13^. The classical 3-step model and quantum mechanical explanations, although good with rare gases, fail to explain properly the HHG in solids such as semiconductors and dielectrics. A more universal model is necessary that takes the linear and non-linear optical properties of solids associated with the Kerr effect into account.

In this paper, an elegant and simple EM Kerr model based on a light wave is presented to explain the SC and HHG generation in different states of matter depending on the response time of the Kerr media. This EM model is an alternative model to the quantum mechanical 3-step model that explains HHG from solids. This work follows the earlier work of Alfano, Hope, and Shapiro based on ESPM^4^. The current model for HHG depends critically on the instantaneous response time of the Kerr media.

Since HHG and SC are nonlinear optical processes, E&M Kerr theory following Bloembergen^18^ and Bloembergen and Shen^19^ explains both HHG and SC generation from an ultrafast optical pulse. The Nonlinear Schrodinger Equation (NLSE) is commonly used to describe the nonlinear optical processes for describing the evolution of the envelope in the slow-varying approximation (SVA) and averaging over many cycles of the nonlinear phase. The NLSE is mainly used to describe the nonlinear effects from supercontinuum arising from self-phase modulation (SPM), four-wave mixing (4WM), cross-phase modulation (XPM), stimulated Raman scattering (SRS), and dispersion effects.

Most recently Alfano et al.^20^ reported on the mechanism of electronic self-phase modulation (ESPM) which follows the instantaneous index of refraction n(t) via n_2_. ESPM arises from the Kerr effect which follows the optical cycles of the phase and envelope of E(t). Kerr in 1875^21^ and Buckingham in 1956^22, 23^ laid down the foundation underlying the mechanism of the change in the index of the refraction on the electric field which can be instantaneous n(t). The changes associated with the electron cloud can be as fast as ~ 50 as (~ 10^–17^ s)–1/3 of the Bohr orbit cycle time. Electronic cloud distortion can be associated with the quiver energy of the electrons. The ESPM mechanism is universal explaining HHG and SC generation in gases, liquids, and solids. The 3-step model^11^ from classical and quantum mechanics for gases fails to explain dielectric and semiconductor solids. Alfano and Shapiro^1^ pointed years before to the electronic cloud mechanism from ESPM in 1970 followed by Alfano et al.^4^ in solids and liquids including Noble gas liquids in 1972.

The electronic cloud mechanism of various materials is believed to have an ultrafast response on the order of 50 as (about 1/3 of Bohr orbital time of 150 as). This instantaneous response leads to HHG arising from fast n_2_^1^. Bloembergen and Yablonovitch^24^ and later Bloembergen^25^ proposed the spectral optical effect from ultrafast ionization plasmas with a sub-picosecond response (10^–13^ s) for the nonlinear Kerr index in materials for the account to broadening and explain more Anti-Stokes broadening observed by Alfano and Shapiro^1–3^ where both electronic and plasma ionization occurs.

Avalanche ionization plasma is a universal mechanism, fast change^24^ which is operative at power flux densities of 10^12^ W/cm^2^ in ionic crystals, glasses, atomic and molecular fluids, and metals. This mechanism influences the plasma index modulation indeed n_2_E^2^ for the spectral broadening by self-phase-modulation. This is a universally observed phenomenon in small-scale filaments in any material observed pronounced broadening when short intense light pulses.

The electronic cloud distortion is based on the Kerr nonlinear index influenced by the carrier-envelope phase and envelope of E(t) at the optical cycle response for noble gases and solids. This mechanism explains the experimental HHG and SC generation from the interaction of high-intensity ultrafast pulses in three states of matter^20^. The ESPM model from nonlinear Kerr index n_2_ reveals three salient features of the HHG: decreasing Harmonics generation followed by a plateau to descending HHG signals to the cutoff frequency. The cutoff frequency can be calculated using the method of the stationary phase on ESPM. This ESPM model is fundamental and an alternative model to the quantum mechanical (QM) three-step model interpretation of HHG. The QM three-step model has difficulty explaining generation in solids. In addition, the ESPM model gives additional features of spectral broadening about the N odd harmonics supporting the theoretical ansatz presented in this paper. Alfano et al.^20^ used a 50-fs laser pulse at intensities of 10^12^ to 10^15^ W/cm^2^ to simulate and experimentally compare the salient features of HHG and supercontinuum about each harmonic from various materials such as gases and solids supporting the Kerr ESPM model. The outcome from the ESPM model is a supercontinuum background superimposed with the sharp odd HHG which was experimentally observed before in various forms of matter.

The pioneers of measuring HHG^6–11^ who started the field with Noble gases seem to miss the much earlier prior art on nonlinear effects for HHG generation by Buckingham^22^ and Alfano and Shapiro^1–3^; and Alfano et al.^4^ papers on effect of n_2_ for noble and solids. Even though the statement on generation of odd harmonics from n_2_ from ESPM is mentioned in ref. 4, the experimental observation of HHG was performed in rare gases in late 1980s and 1990s generated from different lasers systems with durations of 1 ps, 2 ps, 36 ps^26^ and with 120 fs, 410 fs, 760 fs^27^ showed cutoff frequency of HHG a dependence on pulse duration. Balcou et al.^26^ showed HHG at different pump laser intensity and different pulse duration which indicates the significance of physical laser parameters on HHG which supports the parameters used in the electromagnetic model proposed in this paper. Our paper shows the importance of these physical laser parameters as well as the Kerr medium key parameters of the Kerr medium response time and size of n_2_ for HHG and cutoff frequency of HHG which is shown later in theoretical part of the electromagnetic model.

HHG investigators in 1980s–2021 missed n_2_ parameter and its time relaxation dependence (ultrafast, fast, and slow) from Kerr effect to produce HHG. The main underlying mechanism is electronic self-phase modulation (ESPM) from Kerr index^1,4^, and it produces attosecond laser pulse from self-mode locking^20^.

All these HHG researchers seem to be unaware of the mechanism and key parameters of HHG until Corkrum and Levenstein described the 3-step quantum mechanical model based on Fermi’s golden rule for tunnelling and the classical acceleration of the tunneled electron and return to the parent ions for rare gases. Most recently, Reis questioned the quantum mechanical classical model for condensed model for solids and liquids to a more universal model. In 1972, Alfano, Hope and Shapiro using the ESPM EM model showed the HHG can be generated but dropped the terms since the Dewar absorbed the UV frequency^4^. Most recently, Alfano et al.^20^ proposed the EM model to give the salient feature observed in all HHG media to depend on fast ultrafast laser pulses, on n_2_ using EM model from ESPM where media responses to optical cycle from phase and envelope which is universal model depended on n_2_.

In the current paper, the focus is on response time of the n_2_ showing that HHG depends on response time of Kerr n_2_ from electronic clouds in Noble gases and electrons within upper states in condensed media. We focus on the electromagnetic optical pulse model for 50 fs/80 fs and 1 ps laser pulses to simulate the HHG and SC spectra generated using different response Kerr times τ from 100 as to 100 fs for different states of matter: gases, liquids, and solids. The response times of the materials τ used in this simulation for generating HHG are found: (1) the instantaneous electronic cloud response time on the order of 50 as; (2) the fast response time of ionization and molecular redistribution on about 1 fs; and (3) the slower rotational and vibrational relaxation times of 1–10 ps or greater.

## Theory

Following Kerr^21^, Buckingham^22, 23^, Duguay^28^, and Alfano et al.^1, 4^, the general form for the index of refraction based on the index of refraction becomes electric field E dependent:

1 $$n= {n}_{0 }+ {n}_{2}{E(t)}^{2},$$

where n_0_ is the index of refraction, n_2_ is the nonlinear index from various mechanisms, and E is the electric field. Our ansatz is that the index of refraction (n) is a function of angular frequency (ω) and time (t): n(ω,t).

In the EM model, the Kerr index of refraction n_2_ of the material depends on the time response of the underlying mechanisms of the material to the electric field of the laser:${n}_{2}= \sum_{i}{n}_{i}$, where i = mechanisms (such as electronic (~ 10^–17^ s), molecular redistribution (~ 10^–14^ s), plasma (~ 10^–13^ s), rotational (~ 10^–12^ s), librational, and other slower mechanism)^28–32^. The work of Kenney–Wallace showed the various temporal components of the Kerr index in CS_2_^31, 32^.

Based on the Kerr effect, the electric field of the light is distorted in the CEP after an intense light beam propagates a distance z into the material and the electric field of the light has the form:

2 $$\begin{aligned} E\left( {t,\omega } \right) = & E_{0} e^{{ - \frac{{t^{2} }}{{T^{2} }}}} cos\left[ {\phi \left( {t,\omega } \right)} \right] \\ = & E_{0} e^{{ - \frac{{t^{2} }}{{T^{2} }}}} cos\left[ {\omega _{0} t - kz + \varphi } \right], \\ \end{aligned}$$

where the exponential time is T = $\frac{{\tau }_{p}}{\sqrt{2\mathrm{ln}2}}$; τ_p_ is the full width half maximum (FWHM) of the pulse; and the bracket is the phase $\upphi \left(\mathrm{t},\omega \right).$ The phase $\upphi \left(\mathrm{t},\omega \right)$ is modulated by the index of refraction due to Kerr effect. The high laser intensity induces changes in the refractive index from electronic and molecular distortion. The propagation constant becomes time and frequency dependent: $k=\frac{n\omega }{c}$. Expanding about ${\upomega }_{0}$, the modulated instantaneous phase of Carrier Envelope Phase (CEP) under the envelope becomes:

3 $$\phi \left(t,\omega \right)= {\omega }_{0}\left\{t-\frac{n\left(t,\omega \right)z}{c}\right\}+\varphi ,$$

where ${\upomega }_{0}$ is the central angular frequency of the laser, n(t,ω) is the refractive index, z is the propagating distance, and φ is the offset phase (set $\varphi =0$). This phase is the key for the generation of the Supercontinuum and Higher Harmonics where the response time of the material’s index of refraction is critical to the generation of HHG. The offset CEP phase φ is set to be zero for the cosine-like pulse which drives HHG modes. The nonlinear refractive index with quadratic field dependence and the material response time τ is given by:

4 $$n\left(t\right)={n}_{0}+{\int }_{-\infty }^{t}{\int }_{-\infty }^{t}f\left({t}{\prime},{t}^{{\prime}{\prime}}\right)E\left(t-{t}{\prime}\right)E\left(t-{t}^{{\prime}{\prime}}\right)d{t}{\prime}d{t}^{{\prime}{\prime}},$$

where n_0_ is the ordinary index, E the electric field and,

5 $$f\left({t}{\prime},{t}^{{\prime}{\prime}}\right)=\left(\frac{{n}_{2}}{\tau }\right){e}^{- \frac{{t}{\prime}}{\tau }} \delta \left(t-{t}^{{\prime}{\prime}}\right).$$

Here, n_2_ is the nonlinear index and τ is the response time (tau). Equation (4) may be simplified to:

6 $$n\left(t\right)= {n}_{0}+ \left(\frac{{n}_{2}}{\tau }\right){\int }_{-\infty }^{t}{e}^{- \frac{\left(t-{t}{\prime}\right)}{\tau }}{E}^{2}\left({t}{\prime}\right)d{t}{\prime}.$$

The pure electronic mechanism of n_2_ is the instantaneous index of refraction for rare noble gases like Ar, Kr, and Ne and solids involving no translation of nuclei or rotation of atomic cluster. It is expected to have relaxation response time much less than the optical period (<< $\frac{1}{{\omega }_{0}}$), faster than few femtoseconds. For this case, the index n(t) responses to E(t) at optical frequencies. Hence the weighting function ($\frac{1}{\tau }){e}^{- \frac{\left(t-{t}{\prime}\right)}{\tau }}$ may be replaced by $\updelta \left(\mathrm{t}-{\mathrm{t}}^{\mathrm{^{\prime}}}\right)$. Following the Kerr effect, the electronic response of the instantaneous response time on 50 fs nonlinear index for ultrafast laser pulses causes the HHG and responsible for ESPM to become:

7 $${n}_{instantaneous}(t)={n}_{0}+{n}_{2}{[{E}_{0}{e}^{- \frac{{t}^{2}}{{T}^{2}}}cos\phi \left(t\right)]}^{2}.$$

Equation (7) represents the instantaneous response of the index of refraction. This is the ansatz that has been used before in the form of n by luminaries like Kerr^21^ and Buckingham^22^. The ansatz n(t) follows the modulation optical cycles of the phase of E. The instantaneous response is used to follow the optical cycle rather than the envelope of the CEP without time averaging was proposed by Buckingham^22^. On the other hand, the average index of refraction <n(t)> following the envelope of the electric field will reveal the supercontinuum without HHG.

The general form for the nonlinear refractive index with quadratic field dependence is,

8 $$n\left(t\right)={n}_{0}+\delta n= {n}_{0}+ {\int }_{-\infty }^{t}f({t}{\prime},t){E}^{2}\left({t}{\prime}\right)d{t}{\prime},$$

where n_0_ is the normal index, E(t) is the laser electric field assumed to have a Gaussian envelope $({E}_{0}\left(t\right)={E}_{0}{e}^{-\frac{{t}^{2}}{{T}^{2}}})$ and f(t) is the weighting function describing the response of the system; f(t) assumes the form $\frac{{e}^{-\frac{t}{T}}}{\tau }$ where τ is the response time of the material. The incident laser’s electric field has the form given by Eq. (2). Following the ESPM model for the electric field E(t) gives:

9 $$E\left(t\right)={E}_{0}{e}^{- \frac{{t}^{2}}{{T}^{2}}}cos\left(\omega t-\frac{\omega n\left(t\right)z}{c}\right),$$

with the instantaneous n(t):

10 $$n\left(t\right)={n}_{0}+{n}_{2}{[{E}_{0}{e}^{- \frac{{t}^{2}}{{T}^{2}}}cos\left({\omega }_{0}t\right)]}^{2}.$$

Using an averaging procedure on n(t) to simulate the generation of the signal caused by the material response τ resulting in HHG and the associated characteristics that can be found in the experimental results such as the number of odd harmonics and the cutoff frequency.

We have numerically applied an averaging procedure using τ response filter to the n(t) for the Kerr effect in different media:

11 $$<n\left(t,\tau \right)>=\frac{1}{\tau }{\int }_{t}^{t+\tau }n\left({t}{\prime}\right)d{t}{\prime},$$

to generate HHG and SC for the instantaneous electronic cloud response time on about 50 as; the fast response time of ionization and molecular redistribution on about 1 fs; and the slower rotational and vibrational relaxation times of 1–10 ps or greater for different pulse durations.

After substituting Eq. (11), <n(t,τ)> for a response time τ into Eq. (9) to get E(t), the modified electronic self-phase modulated spectral E(ω) is obtained by the Fast Fourier Transform (FFT) technique. The spectral density S(ω) of the phase-modulated light at ω is:

12 $$S\left(\omega \right)=\frac{c}{4\pi }{\left|E(\omega )\right|}^{2},$$

where E(ω) is the Fourier transform of E(t).

For a material with response time τ slower than pure electronic on the order of such as molecular redistribution, plasma, librational, orientational, and vibrational motion $(t>\tau >\frac{10}{{\omega }_{L}})$, the envelope of the pulse is reflected in n(t) and no HHG is produced. For slow response time of the material τ, the average n(t) becomes the “classical” SPM following the envelope of the pulse:

13 $${n}_{slow}\left(t\right)={n}_{0}+\frac{{n}_{2}}{2}{\left[{E}_{0}{e}^{- \frac{{t}^{2}}{{T}^{2}}}\right]}^{2}.$$

## Results

**Figure 1:**
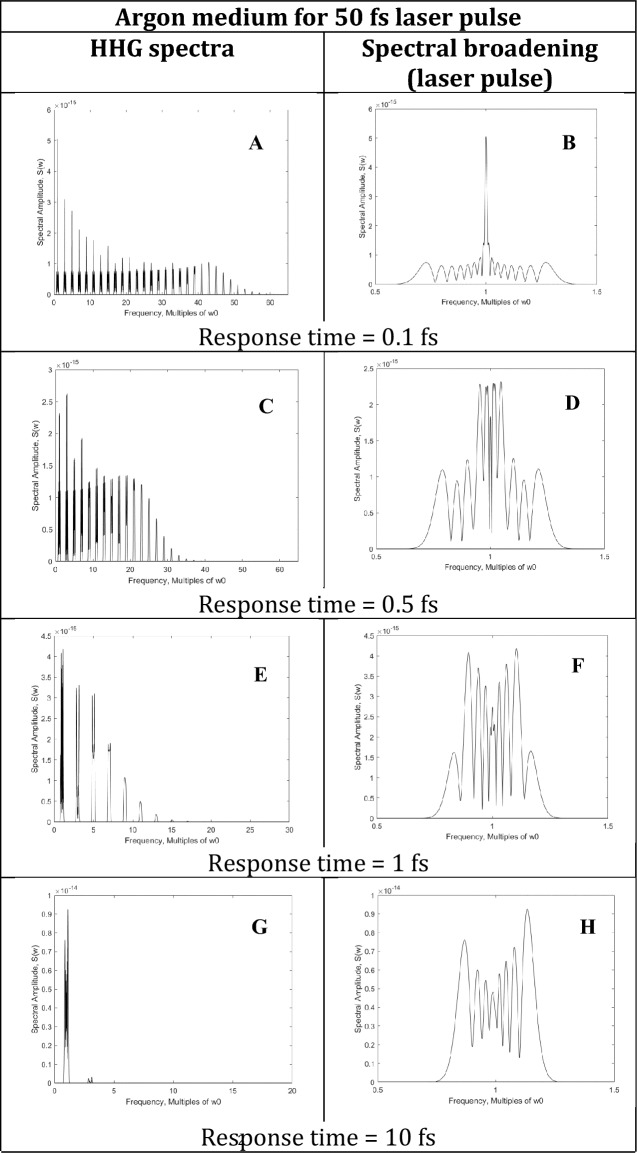
Argon (n_0_ = 1 and n_2_ = 2.5 × 10^–19^ cm^2^/W) HHG spectra (**A**,**C**,**E**,**G**) and spectral broadening of laser frequency (**B**,**D**,**F**,**H**) due to different response times of the propagating medium (0.1 fs for **A** and **B**; 0.5 fs for **C** and **D**; 1 fs for **E** and **F**, and 10 fs for **G** and **H**) for visible laser pulses with wavelength = 500 nm (ω_0_ = 2.48 eV) and an optical cycle, T = 1.67 fs, pulse duration = 50 fs, pulse energy = 5 mJ, laser spot size = 20 µm, and medium propagation distance = 0.5 mm.

The simulations on the effects on HHG and SC spectra for the Kerr response time for gases and solids are shown in Fig. 1 and in the supplement figures. It is shown that the ultrafast and fast Kerr response times τ from the ultrafast on the order of 100 as (0.1 fs) for electronic cloud distortion to fast 1 fs for plasma formation and 10 fs for molecular redistribution affect the number of odd harmonics in HHG and the extend of the broadening of the pump laser from SPM.

The spectra have been simulated using a visible laser pulse at 500 nm and a NIR pulse at 800 nm, 1600 nm, and 1240 nm in MATLAB-based computer simulation code from the Kerr index model (Eqs. 9–12). These HHG and SC spectra are displayed in Fig. 1 for Ar at 500 nm and in the Supplement figures for Ar at 800 nm, ZnO at 1600 nm, and LBG at 1240 nm with various Kerr parameters and Kerr response times.

Figure 1 shows the HHG spectra and the spectral broadening around the laser frequency for different response time τ from an Argon (n_0_ = 1 and n_2_ = 2.5 × 10^–19^ cm^2^/W) gas medium while the laser pulse propagates through the medium for 0.5 mm. The laser pulse used here has a visible wavelength of 500 nm with an optical cycle, T = 1.67 fs, a pulse duration of 50 fs, a pulse energy of 5 mJ, and a laser spot size of 20 µm for a peak intensity of 3.18 × 10^16^ W/cm^2^. Each pair of figures in Fig. 1 represent HHG spectrum and laser frequency broadening for different response times: Fig. 1A,B for 0.1 fs, Fig. 1C,D for 0.5 fs, Fig. 1E,F for 1 fs, and Fig. 1G,H for 10 fs response time of the medium. The change in the index of refraction caused by the Kerr effect from the intense pulses is less than the ordinary index n_0_. At higher intensities, n_4_ needs to be included for ultra-supercontinuum (USC)^33^.

The instantaneous Kerr index creates 61 harmonics (61ω_0_) as the cut-off frequency in Fig. 1A yielding 8.2 nm in the X-ray region. The higher harmonics generated for the slower Kerr index reduce sharply shown in Fig. 1C,E,G: 39ω_0_, 17ω_0_, and 3ω_0_, respectively. This behavior in the HHG cut-off is supported by other materials shown in the supplement.

The HHG generation shown in Fig. 1 displays the effect of different response times τ of the Argon Kerr medium for the interacting laser pulses with 50 fs. Figure 1A shows the HHG spectrum generated due to the very fast ESPM interaction at 0.1 fs (100 as) response time. The HHG spectrum arising from EPSM generates the characteristic features of odd harmonics: decreasing harmonics, plateau, and cut-off frequency^4, 15^.

Figure 1E shows the spectrum with the odd harmonics due to the fast 1 fs response time in comparison to the instantaneous response of 0.1 fs (100 as) from the electronic response. This spectrum has odd harmonics with a cutoff frequency ending at the17th harmonic which is much shorter than Fig. 1A (cut-off ending at the 61st harmonic). Figure 1F shows the spectral broadening to the laser frequency which has a shorter spectral broadening than Fig. 1B with higher intensities. For the response time τ = 1 fs of the interacting material, both ESPM and SPM dominate for a 50-fs laser pulse due to the fast response time of ionization and molecular redistribution, and a hint of HHG spectrum can be observed with high-intensity spectral broadening of laser frequency from 50 fs input pulse duration.

For a slower 10 fs Kerr medium response time, Fig. 1G shows the decreasing odd harmonics with an even shorter spread (ending at 1st harmonic) from the 50 fs input laser pulses. Figure 1H shows spectral broadening around the laser frequency even shorter in spread but with higher intensity. For the response time τ = 10 fs of the interacting material, SPM dominates for a 50-fs laser pulse due to the slower rotational and vibrational relaxation times of 1–10 ps or greater.

Additional HHG spectra and experimental comparison spectra are shown in the supplement, Fig. S1 shows HHG spectra and laser frequency spectral broadening is produced for Argon medium from different medium response times of 2 fs, 4 fs, 6 fs, 8 fs, and 100 fs for the same laser pulse with a pulse duration of 50 fs. Compared to Fig. 1 with a shorter pulse duration of 50 fs, Supplement Fig. S2 is for HHG and SC with a pulse duration of 1 ps shows a drastic reduction of both the number of Higher Harmonics and the laser frequency spectral broadening which indicates the fast and slow laser-medium interaction, respectively. Additional supporting data is shown in the supplement Figs. S3–S8 on HHG spectra and laser frequency spectral broadening is simulated for different media such as Ar at 800 nm with an optical cycle, T = 2.67 fs (simulating Spectra-Physics Solstice laser system with Pulse energy of 5 mJ, wavelength of 800 nm, and pulse duration of 80 fs), ZnO at 1600 nm with an optical cycle, T = 5.33 fs and Lead–Bismuth–Gallium oxide glass (LBG glass) at 1240 nm with an optical cycle, T = 4.13 fs at the pulse duration of 50 fs and 1 ps for different Kerr response time. The laser pulse parameters are adjusted based on experimental results from the literature.

In Fig. S12 in the supplement, the experimental result shows the generation of even higher harmonics from MoS_2_. This demonstrates that the 3-step model of Lewenstein et al. cannot explain the even harmonic generation. However, the ESPM model can explain the even higher harmonic generation by incorporating n_1_E terms. These even harmonics can be explained by the n1 term in the refractive index: n = n_0_ + n_1_E + n_2_E^2^ into the phase, adding n_1_ term in Eq. (1). Our future research will investigate the effect of n_1_ term in the refractive index equation to produce the even harmonics. Work has been done in the past with materials with χ^2^ and χ^3^ even in the original Alfano and Shapiro paper in glass and later in calcite and quartz^1–3^.

## Discussion

**Figure 2:**
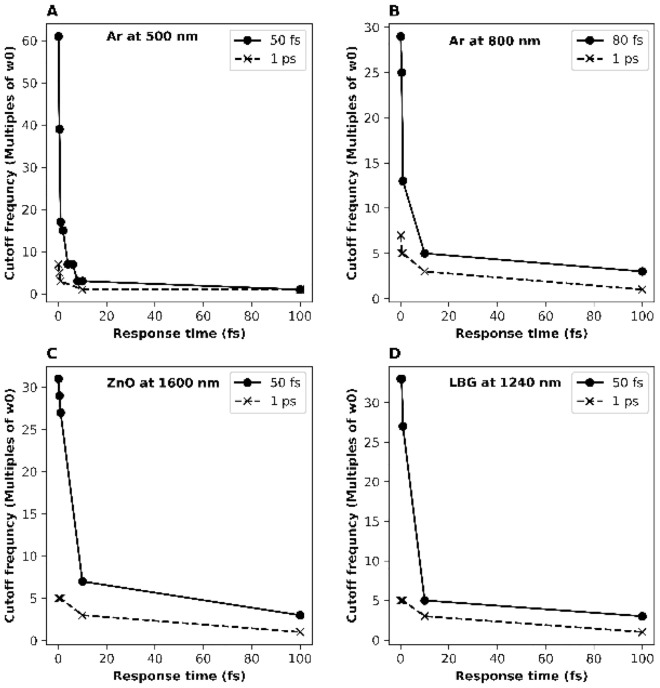
Cutoff frequency in multiples of laser frequency, ω_0_ vs the response time, τ in fs for different materials: (**A**) for Argon with 500 nm laser beam; (**B**) for Argon with 800 nm laser beam; (**C**) for ZnO with 1600 nm laser beam; and (**D**) for LBG glass with 1240 nm laser beam. The input laser pulses for each of the subplot have pulse durations of 50 fs (·) and 1 ps (x).

The outcome of the number of odd harmonics from Fig. 1 is shown in Fig. 2 along with other materials simulated in the supplement. The number of odd HHG pulses (N) and the cutoff frequency vs the response time of the Kerr material is displayed in Fig. 2 for Argon, ZnO, and Lead–Bismuth–Gallium oxide (LBG) glass for fs and ps pulses. The number of harmonics N shown depends critically on the response time (τ) of the material on the order of the optical cycle of 1 fs. The salient feature that is shown in Fig. 2 is the rapid decrease of N with longer response time showing the importance of ESPM from electronic cloud distortion. For the 50-fs input pulse, the number harmonics reduces for the slower response time from 61 harmonics for 0.1 fs, 39 harmonics for 0.5 fs, 17 harmonics for 1 fs, and 3 harmonics for 10 fs. For the 1 ps input pulse, the number harmonics reduces for the slower response time from 7 harmonics for 0.1 fs to 1 harmonic for 10 fs. These results clearly demonstrate that the instantaneous response of a material from ESPM on the order of an optical cycle (1.67 fs for a 500 nm pulse) generates the greatest number of HHG and longest cut-off frequency for HHG which depends on electronic cloud distortion for the Kerr effect present in materials such as rare gases and solids. Moreover, the number of odd harmonics reduces significantly when the response time of the medium is larger than the optical cycle T. Figure 2A with the fastest optical cycle T = 1.67 fs (500 nm) shows the fastest drop of cutoff frequency and Fig. 2C with the slowest optical cycle T = 5.33 fs (1600 nm) shows the slowest drop of cutoff frequency over response time τ. A broader analysis of the supplement figures will show how HHG structure disappears when the response time τ is a few times larger than the optical cycle. A comparison between recent experimental results of HHG from solids and theoretical predictions from the EM based ESPM model discussed in this paper is shown at Figs. S9–S12 in the supplement using wavelengths in optical, NIR, and MIR regimes^20^.

## Conclusion

In conclusion, the electromagnetic (EM) optical model simulates the HHG and SC spectra generated from 50 fs pulse and a 1 ps pulse for different response Kerr times τ for different states of matter: gases, liquids, and solids from the instantaneous ultrafast electronic cloud response on about 100 as. A fast response of molecular redistribution and plasma times on about 1 fs for HHG. and SC. The slower rotational and vibrational relaxation times of 10 ps or greater gives only SC and not the HHG. It’s critical to have a < 100 fs pulse duration for substantial HHG generation for the response time on the order of the optical cycle. This work supports a universal, elegant, and simple Kerr EM theoretical model^1, 4^ fitting HHG from different states of matter and different pump wavelengths in optical, NIR, and MIR regimes. The EM model is an alternative model to the quantum mechanical 3-step model for HHG using femtosecond pulses on its three characteristic features including the cut-off frequency for different states of matter and should be the theory of choice in the HHG field. This work on EM model was presented at APS March Meeting 2023 in person^34^.

A new fundamental electromagnetic wave equation needs to be developed from Maxwell’s equations including the instantaneous response for both the envelope and the phase of the optical light pulse from nonlinear polarizability. This universal EM model will be in lieu of the current nonlinear Schrodinger equation (NLSE) based on the slowly varying envelope approximation (SVEA) and the time-dependent semi-classical quantum–mechanical 3-step model. The EM model needs to respond to the index instantaneously to explain HHG as a process being driven and created coherently from the optical pump. The EM model can also explain the general of attosecond pulses from the Kerr index^20^.

### Supplementary Information

Supplementary Information.

## Data Availability

Data underlying the results presented in this paper are not publicly available at this time but may be obtained from the corresponding author upon reasonable request.
