# Supplementary material for: Higher harmonics and supercontinuum generated from the Kerr response time in different states of matter from a universal electromagnetic model
Source: Sci Rep. 2023 Sep 19;13:15467. doi: 10.1038/s41598-023-42579-z (PMC10509186; doi:10.1038/s41598-023-42579-z)
Supplement: Supplementary file 1 — Supplementary Information. [file 41598_2023_42579_MOESM1_ESM.docx]

**Supplement for:**

**“Higher Harmonics and Supercontinuum Generated from the Kerr Response Time in Different States of Matter from a Universal Electromagnetic Model”**

Robert R. Alfano and Shah Faisal B. Mazhar

CUNY Institute for Ultrafast Spectroscopy and Lasers,

Physics Department,

City College of New York, New York, NY 10031

**Supplementary spectral figures for HHG and Supercontinuum in different materials and comparison with experimental HHG data on solids**

The supplement Fig. S1 to Fig. S8 contain HHG spectra and SC spectra from various states of matter for different Kerr response times: Argon at visible 500 nm and 50 fs and 1 ps and at NIR 800 nm at 80 fs and 1 ps; ZnO 1600 nm at 50 fs and 1 ps; and LBG at 1240 nm at 50 fs and 1 ps. The salient generation of the HHG arises from the cos(cos^2^) in E(t) to form odd Bessel function, reference [S1].

Figs. S1 to S8 show the same reduction of the number of odd harmonics N as the response time of the medium gets slower for femtosecond input laser pulses for various response time of Argon, ZnO, and LBG media which is shown in Fig. 2 in the manuscript. For a 1 ps input laser pulses, N reduces significantly more, not showing any significant ESPM or SPM signature as described in the manuscript. This outcome demonstrates the critical nature of the response time of the various states of matter to the nonlinear index of refraction n_2_ for HHG and SC generation.

Figs. S9 to S12 show recent experimental results of HHG from solids like ZnO, MgO, and MoS_2_ respectively along with the theoretical prediction from the presented ESPM model discussed in the manuscript. These figures show comparison between the experimental result and theoretical prediction in terms of the cutoff frequency and HHG structures.

In Fig. S12, the even harmonics are generated in the HHG spectrum from the uniaxial MoS_2_. This even higher harmonics demonstrates that the electromagnetic model can be used to explain the generation of even harmonics while the quantum mechanical 3-step model cannot [S2]. We plan to investigate in the future research including the n_1_ term. This even harmonic further supports the EM ESPM model where the n=n_0_+n_1_E+n_2_E^2^ into the phase. In the past, Alfano and Shapiro worked with materials with χ2 and χ3 even in the original papers in glass and later in calcite and quartz [S1, S3-S5].

| **HHG spectra** | **Spectral broadening of the laser pulse** |
| --- | --- |
| 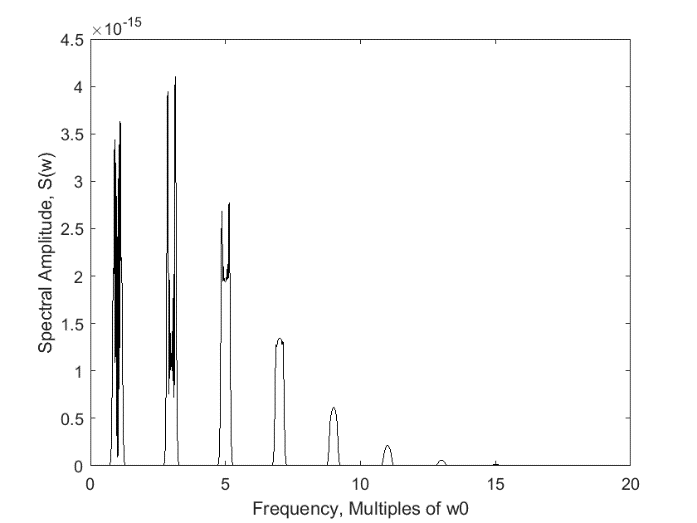  **A** | 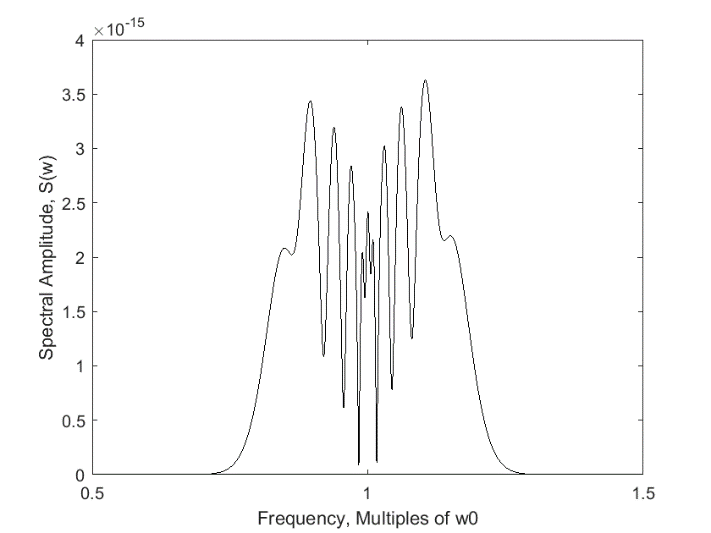  **B** |
| Reseponse time = 2 fs | |
| 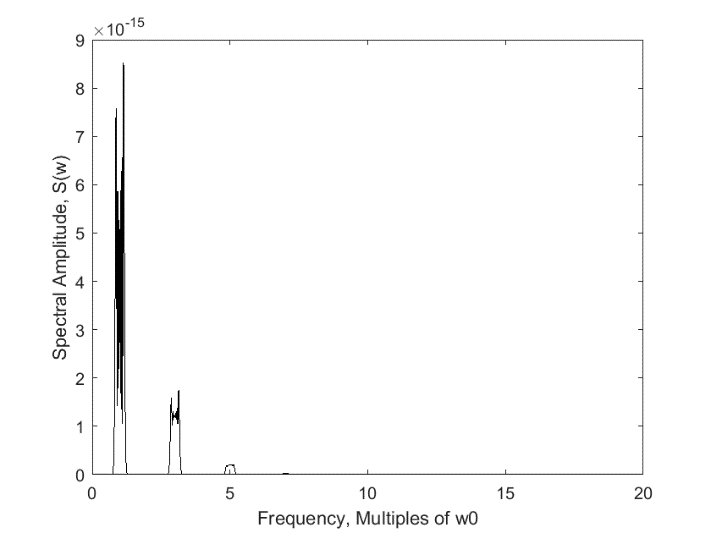  **C** | 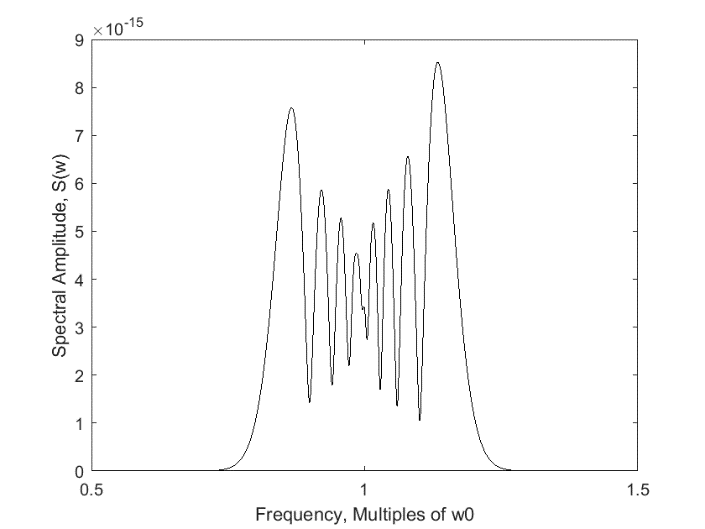  **D** |
| Reseponse time = 4 fs | |
| 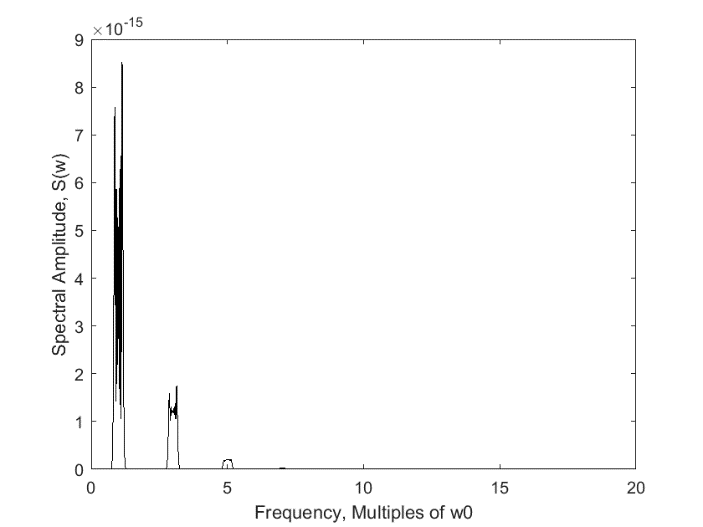  **E** | 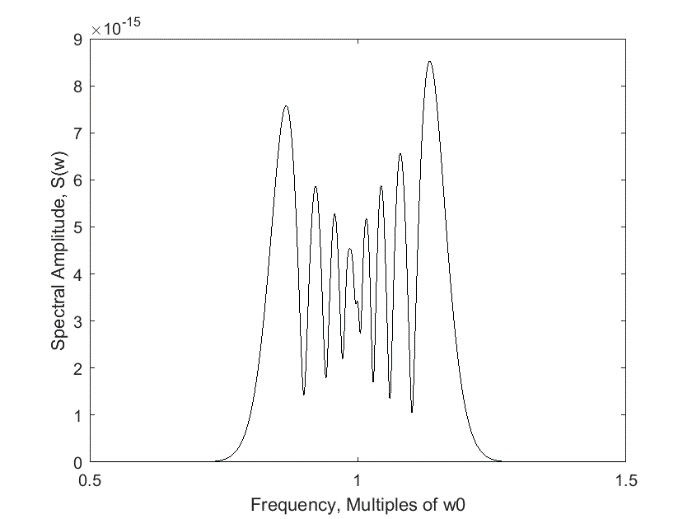  **F** |
| Reseponse time = 6 fs | |
| 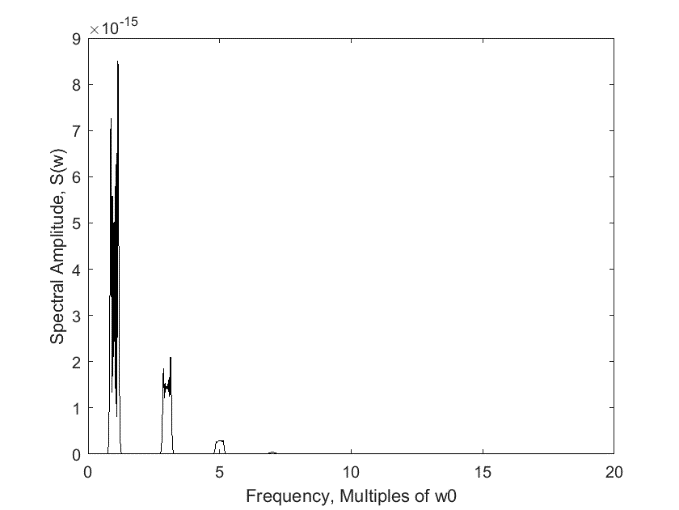  **G** | 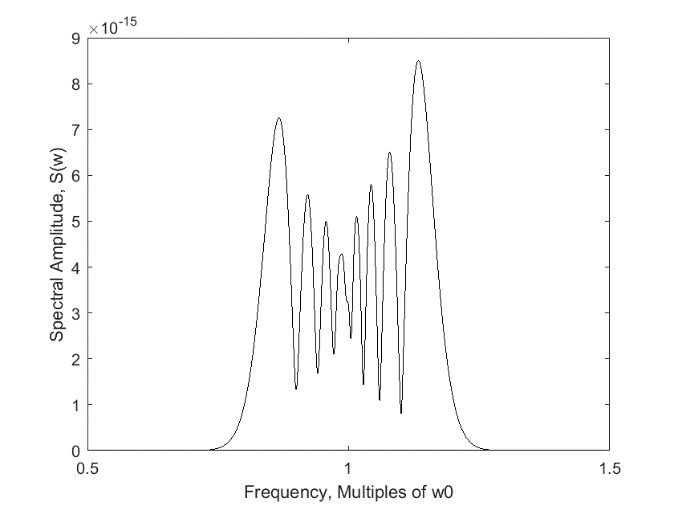  **H** |
| Reseponse time = 8 fs | |
| 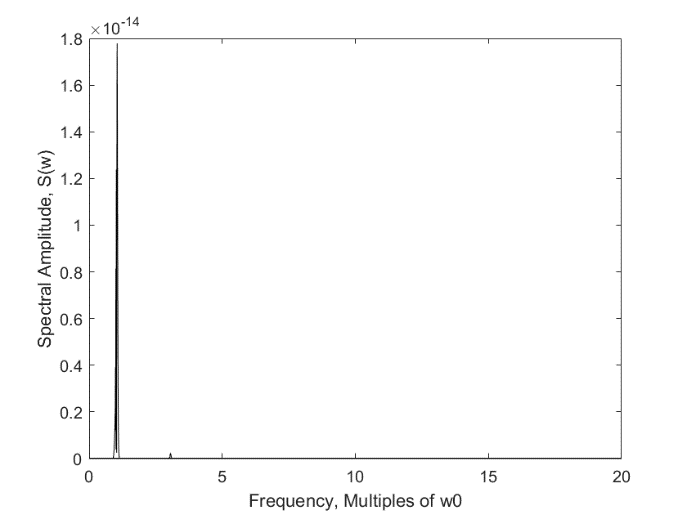  **I** | 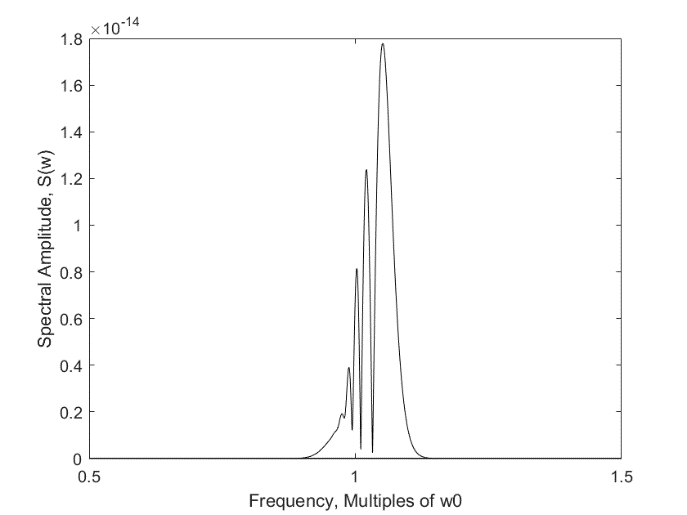  **J** |
| Reseponse time = 100 fs | |
| **Fig-S1:** HHG spectra (A,C,E,G,I) and spectral broadening of laser frequency (B,D,F,H,J) due to different response times of the propagating medium (2 fs for A and B; 4 fs for C and D; 6 fs for E and F, 8 fs for G and H, and 100 fs for I and J) for **Argon** medium (n_0_ = 1 and n_2_ = 2.5x10^-19^ cm^2^/W) and the laser pulses with wavelength = 500 nm, **pulse duration = 50 fs**, pulse energy = 5 mJ, laser spot size = 20 µm, and medium propagation distance = 0.5 mm. | |

| **HHG spectra** | **Spectral broadening of the laser pulse** |
| --- | --- |
| 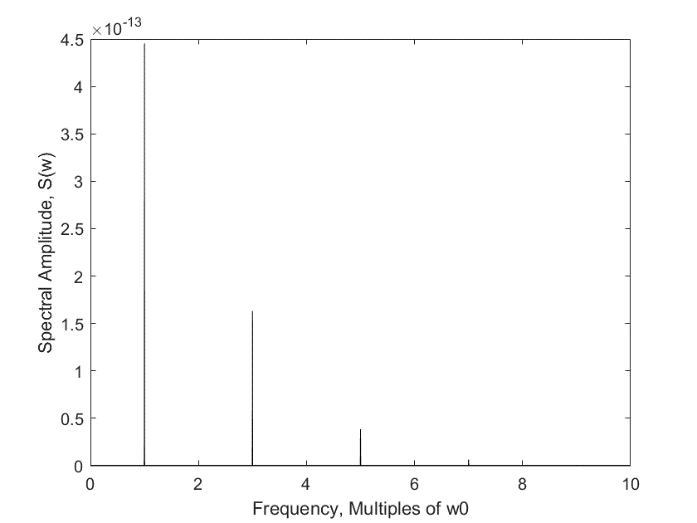  **A** | 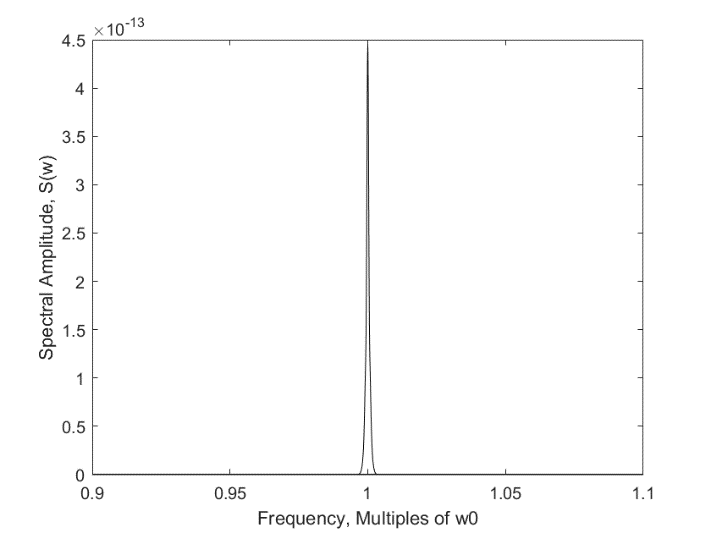  **B** |
| Reseponse time = 0.1 fs | |
| 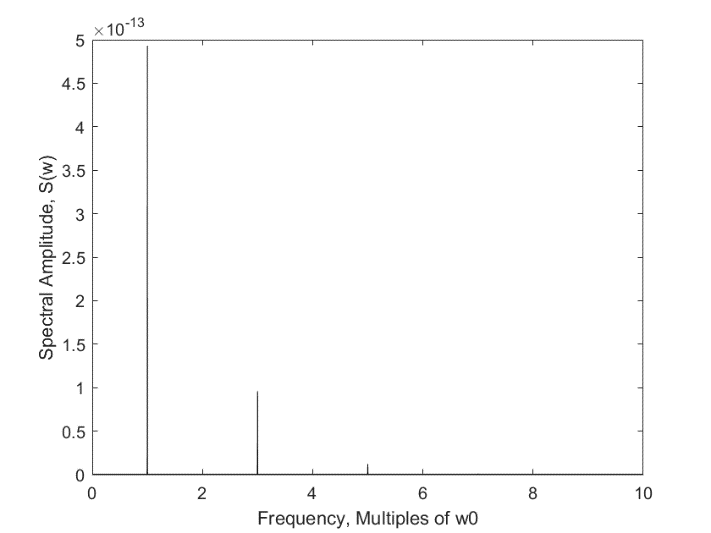  **C** | 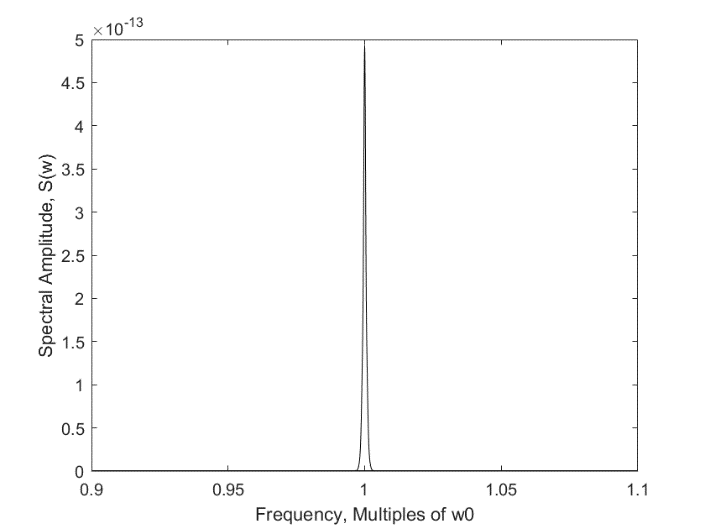  **D** |
| Reseponse time = 0.5 fs | |
| 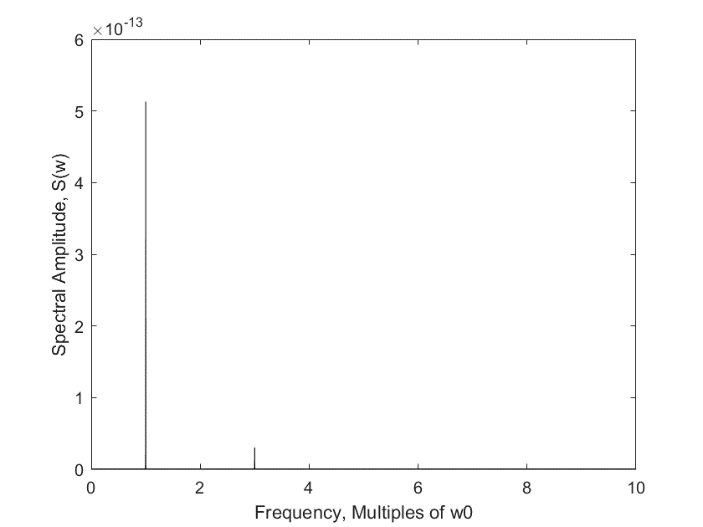  **E** | 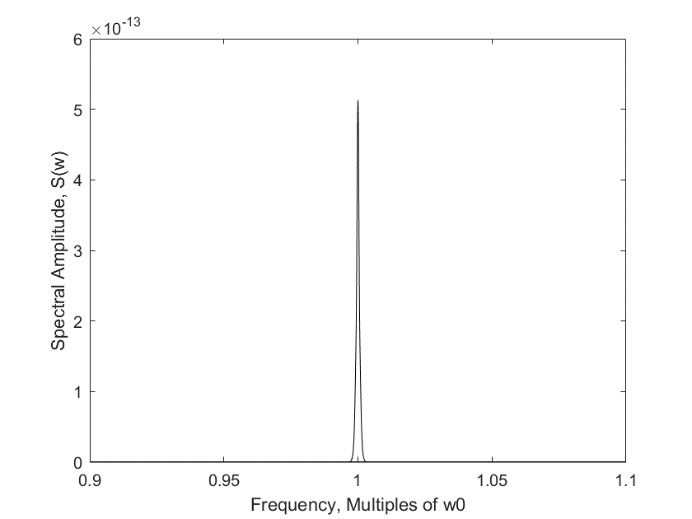  **F** |
| Reseponse time = 1 fs | |
| 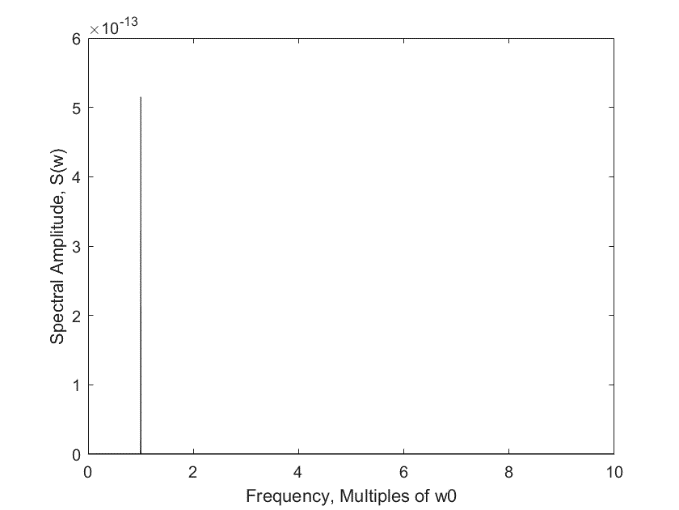  **G** | 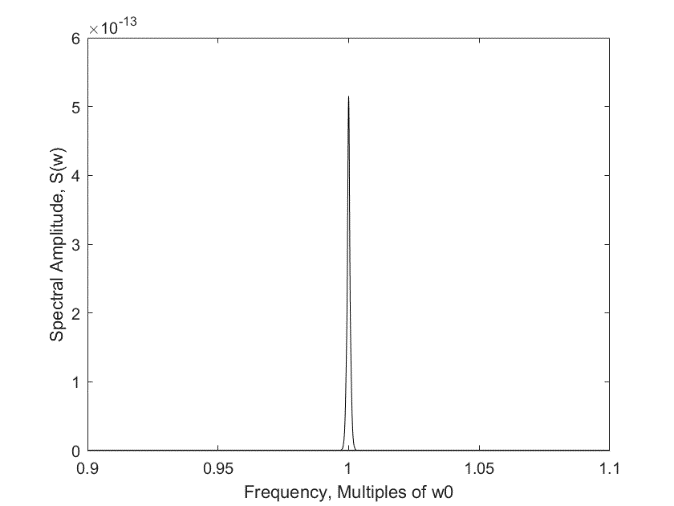  **H** |
| Reseponse time = 10 fs | |
| 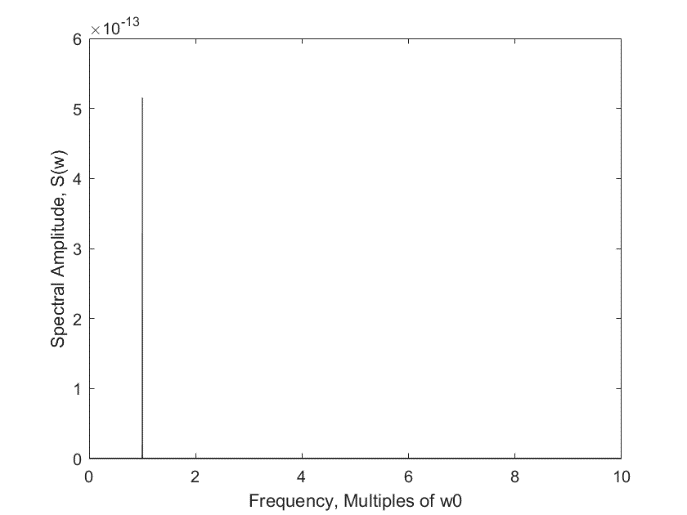  **I** | 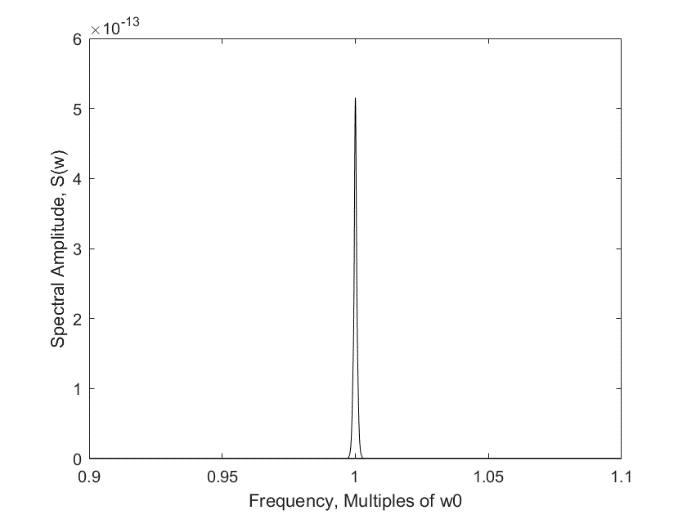  **J** |
| Reseponse time = 100 fs | |
| **Fig-S2:** HHG spectra (A,C,E,G,I) and spectral broadening of laser frequency (B,D,F,H,J) due to different response times of the propagating medium (0.1 fs for A and B; 0.5 fs for C and D; 1 fs for E and F, 10 fs for G and H, and 100 fs for I and J) for **Argon** medium (n_0_ = 1 and n_2_ = 2.5x10^-19^ cm^2^/W) and the laser pulses with wavelength = 500 nm, **pulse duration = 1 ps**, pulse energy = 5 mJ, laser spot size = 20 µm, and medium propagation distance = 0.5 mm. | |

| **HHG spectra** | **Spectral broadening of the laser pulse** |
| --- | --- |
| 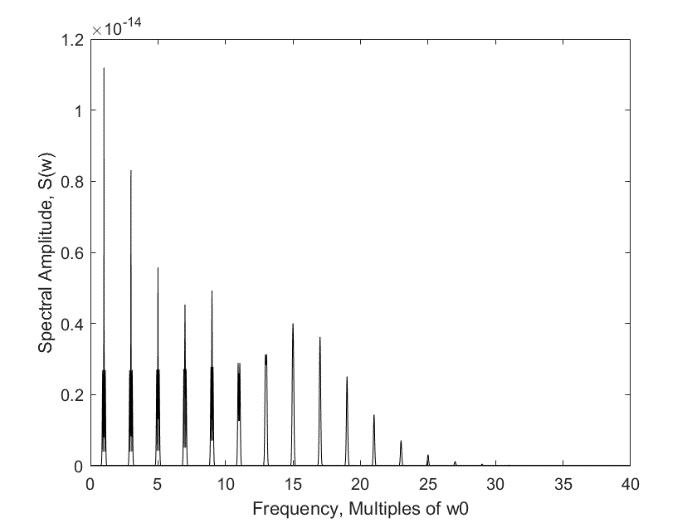  **A** | 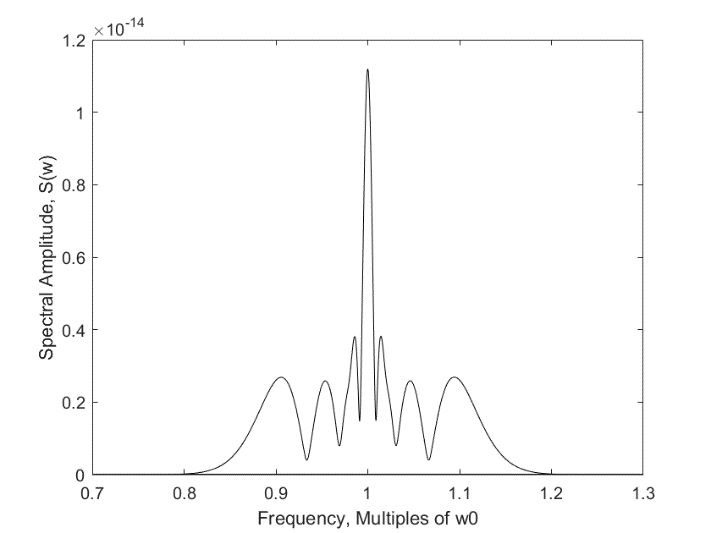  **B** |
| Reseponse time = 0.1 fs | |
| 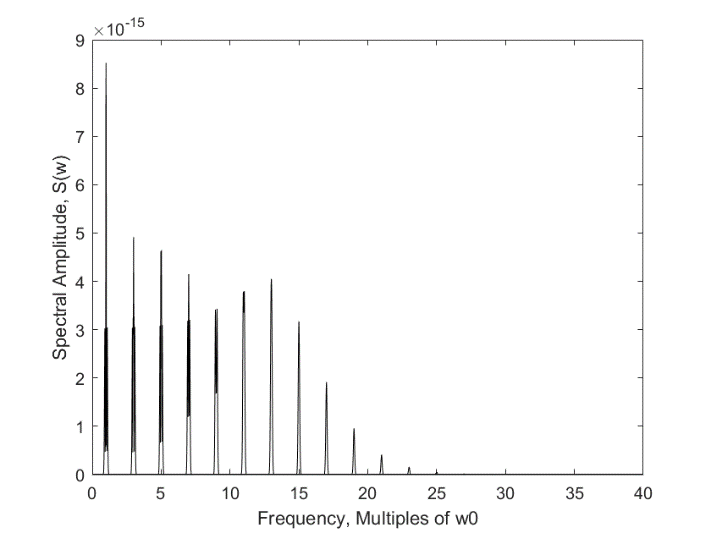  **C** | 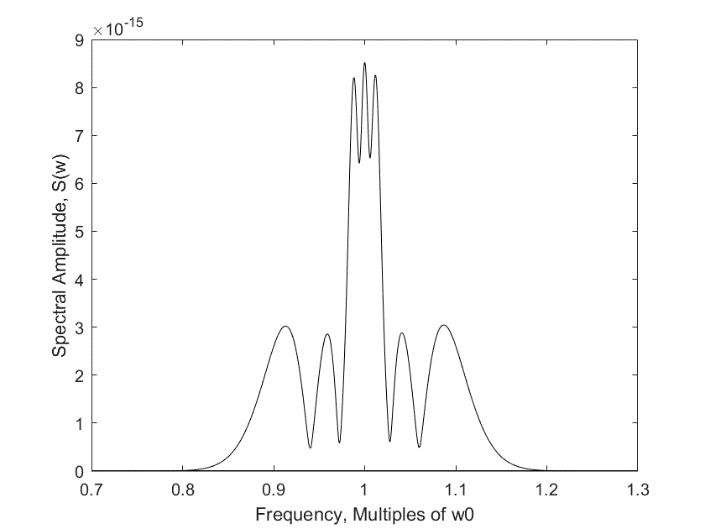  **D** |
| Reseponse time = 0.5 fs | |
| 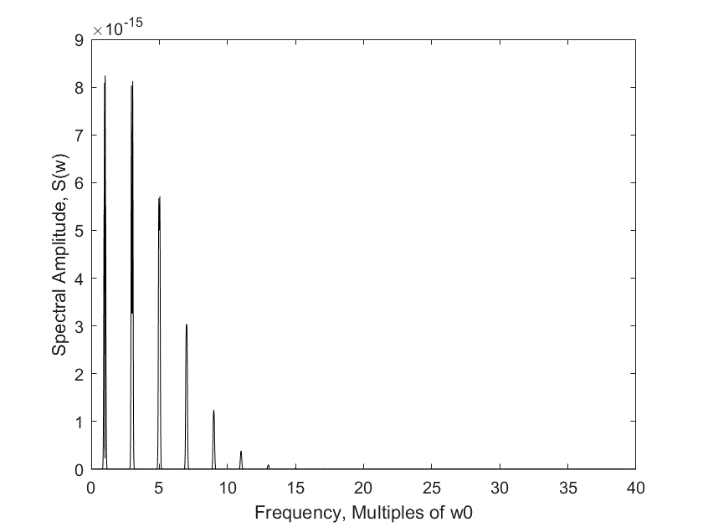  **E** | 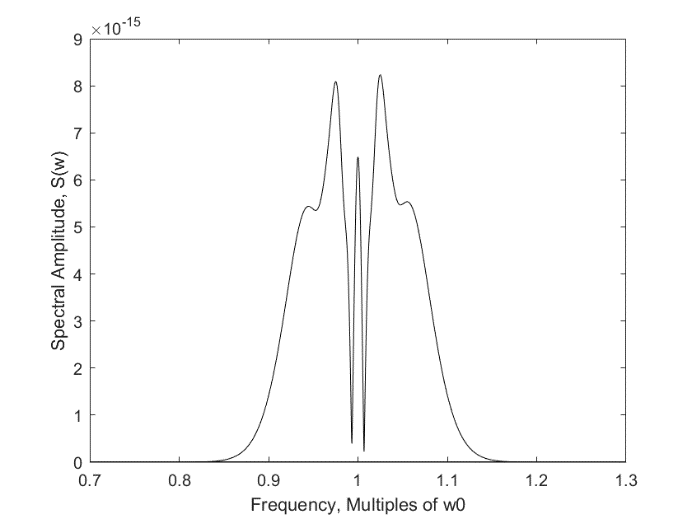  **F** |
| Reseponse time = 1 fs | |
| 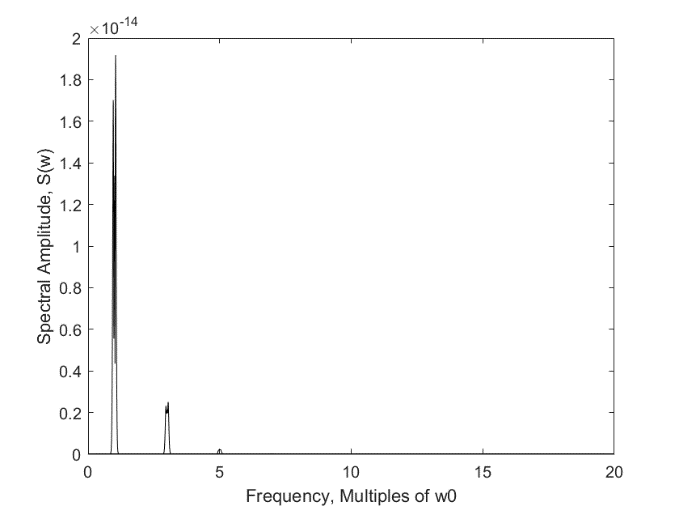  **G** | 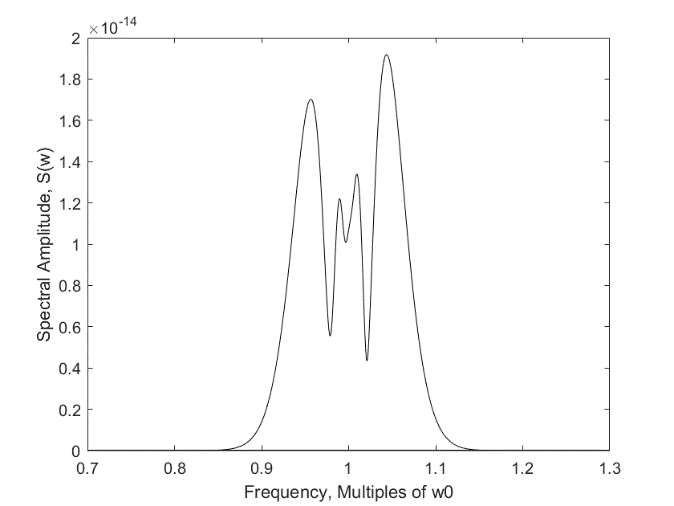  **H** |
| Reseponse time = 10 fs | |
| 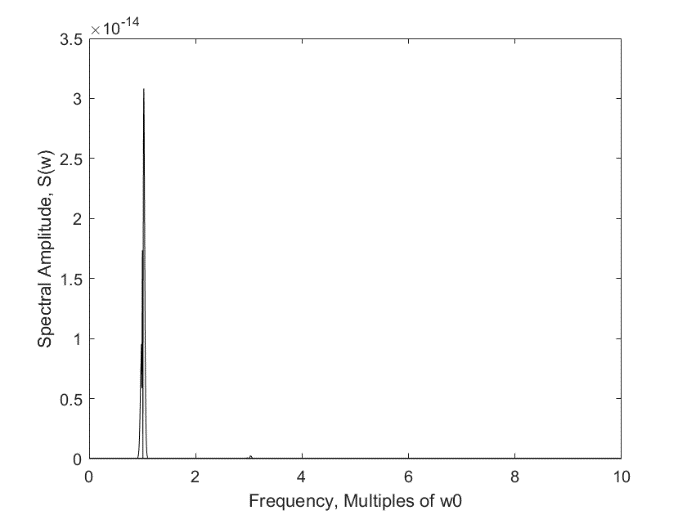  **I** | 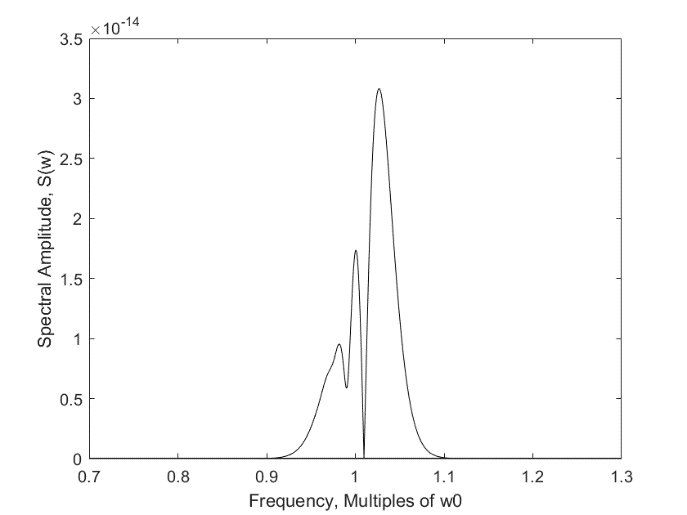  **J** |
| Reseponse time = 100 fs | |
| **Fig-S3:** HHG spectra (A,C,E,G,I) and spectral broadening of laser frequency (B,D,F,H,J) due to different response times of the propagating medium (0.1 fs for A and B; 0.5 fs for C and D; 1 fs for E and F, 10 fs for G and H, and 100 fs for I and J) for **Argon** medium (n_0_ = 1 and n_2_ = 2.5x10^-19^ cm^2^/W) and the laser pulses with **wavelength = 800 nm**, **pulse duration = 80 fs**, pulse energy = 5 mJ, laser spot size = 20 µm, and medium propagation distance = 0.5 mm. | |

| **HHG spectra** | **Spectral broadening of the laser pulse** |
| --- | --- |
| 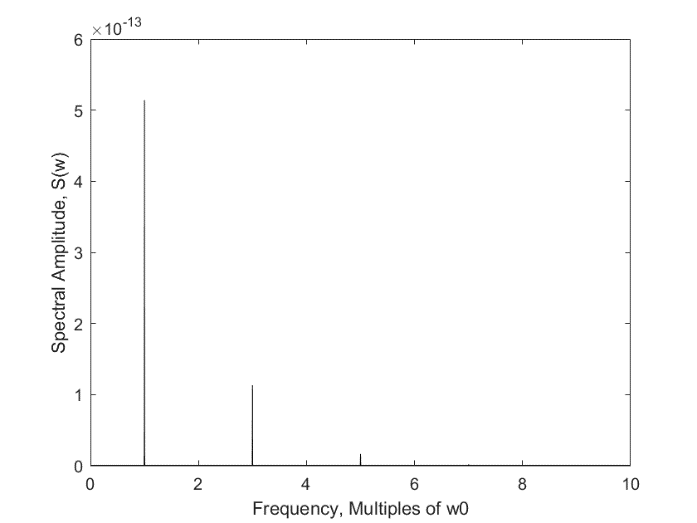  **A** | 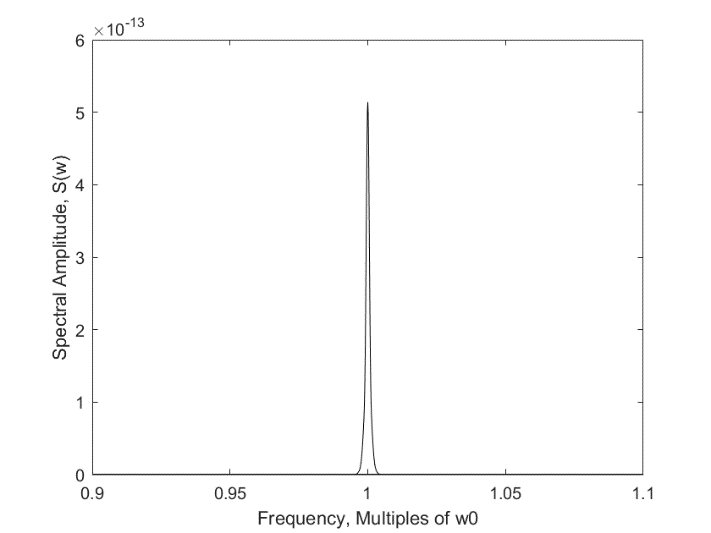  **B** |
| Reseponse time = 0.1 fs | |
| 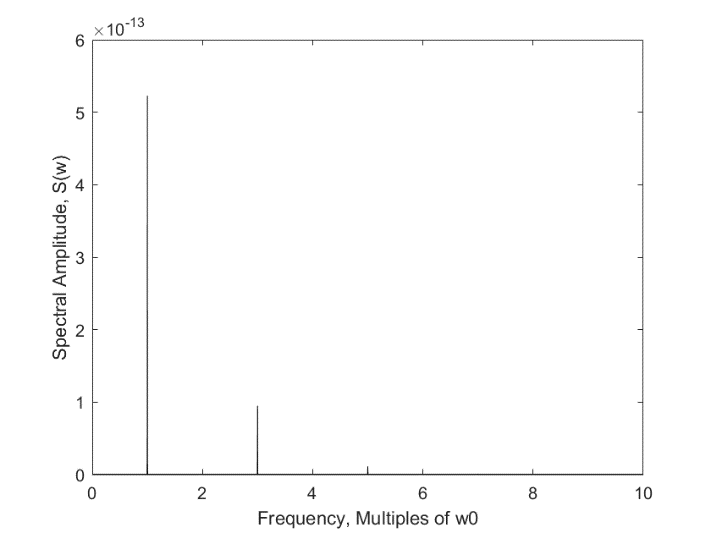  **C** | 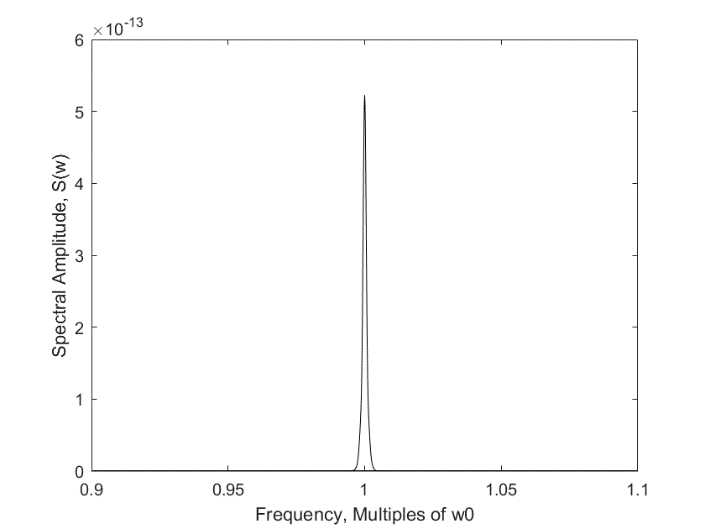  **D** |
| Reseponse time = 0.5 fs | |
| 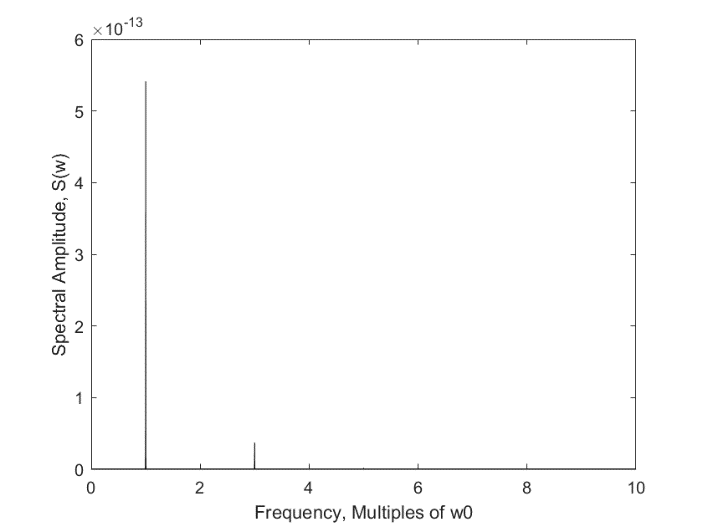  **E** | 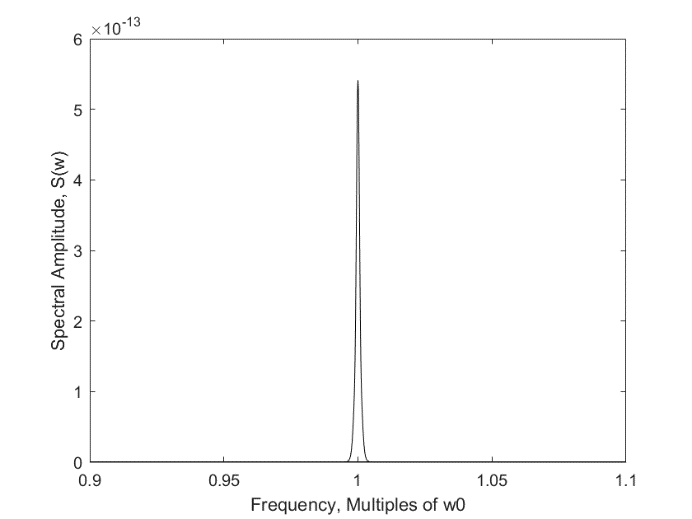  **F** |
| Reseponse time = 1 fs | |
| 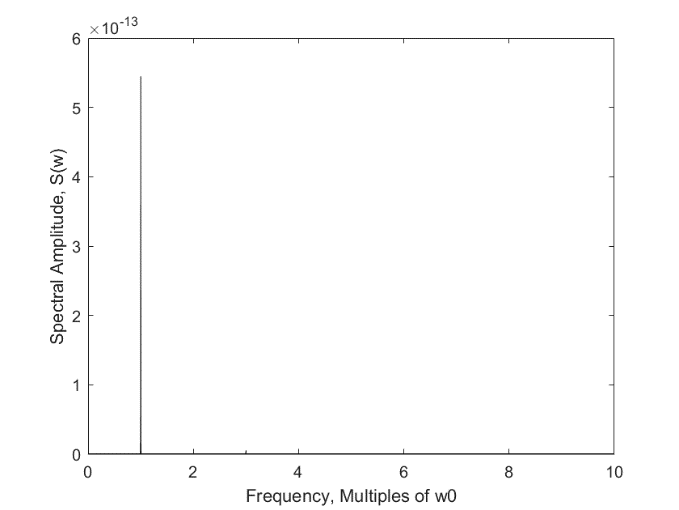  **G** | 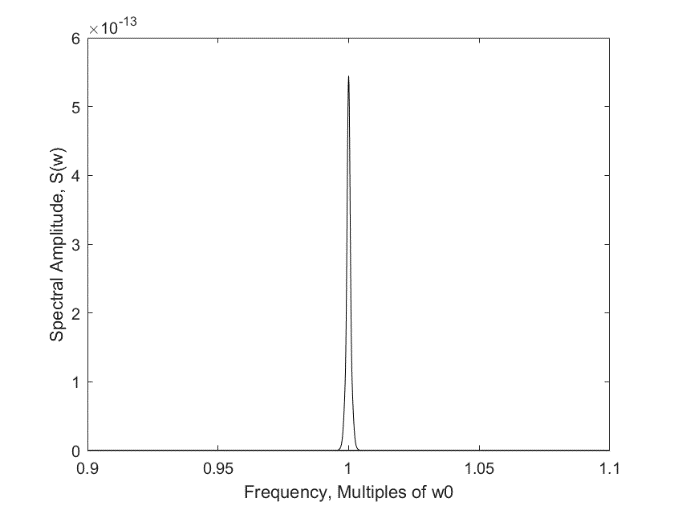  **H** |
| Reseponse time = 10 fs | |
| 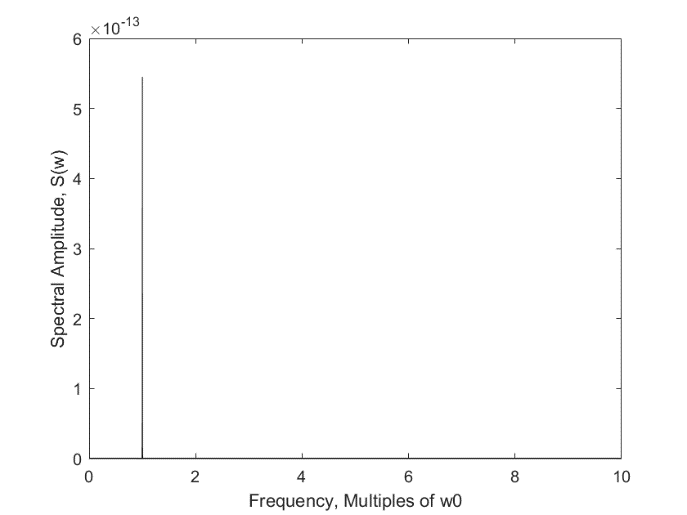  **I** | 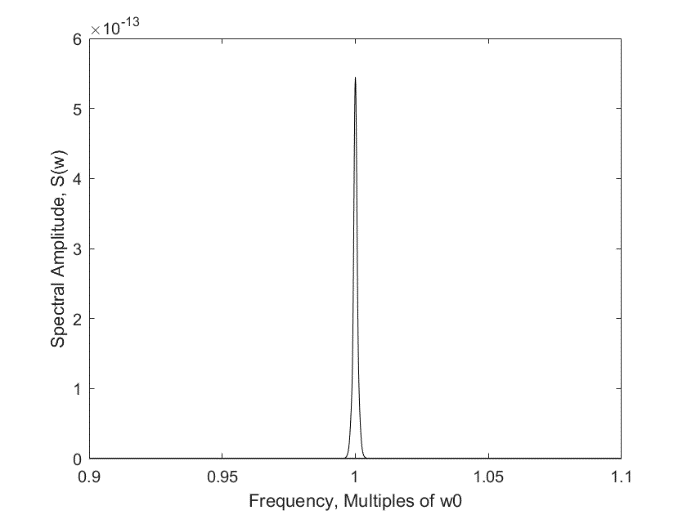  **J** |
| Reseponse time = 100 fs | |
| **Fig-S4:** HHG spectra (A,C,E,G,I) and spectral broadening of laser frequency (B,D,F,H,J) due to different response times of the propagating medium (0.1 fs for A and B; 0.5 fs for C and D; 1 fs for E and F, 10 fs for G and H, and 100 fs for I and J) for **Argon** medium (n_0_ = 1 and n_2_ = 2.5x10^-19^ cm^2^/W) and the laser pulses with **wavelength = 800 nm**, **pulse duration = 1 ps**, pulse energy = 5 mJ, laser spot size = 20 µm, and medium propagation distance = 0.5 mm. | |

| **HHG spectra** | **Spectral broadening of the laser pulse** |
| --- | --- |
| 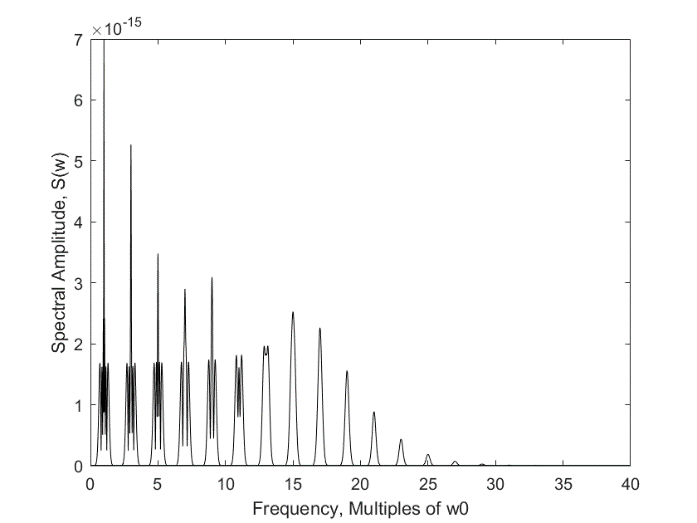  **A** | 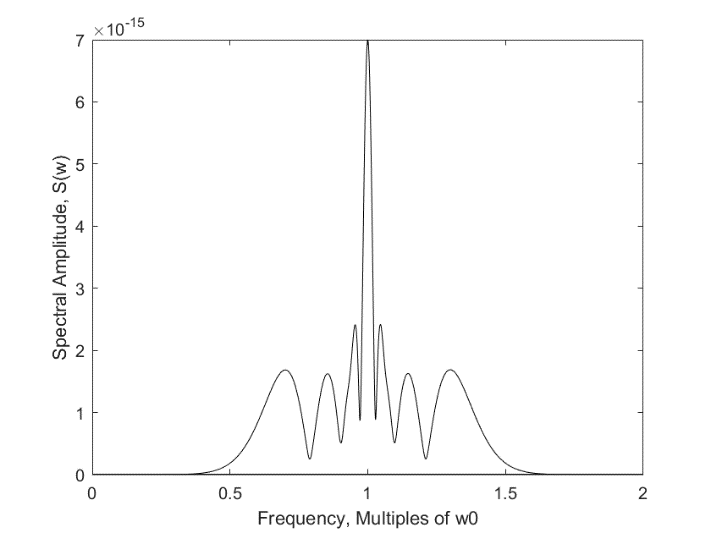  **B** |
| Reseponse time = 0.1 fs | |
| 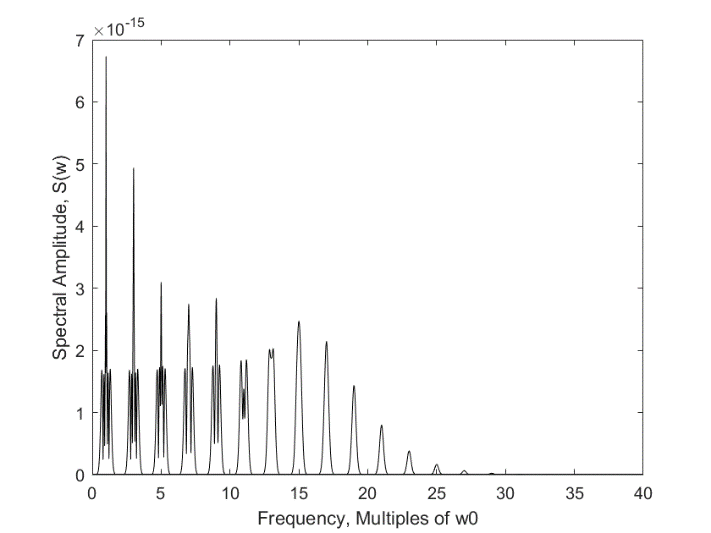  **C** | 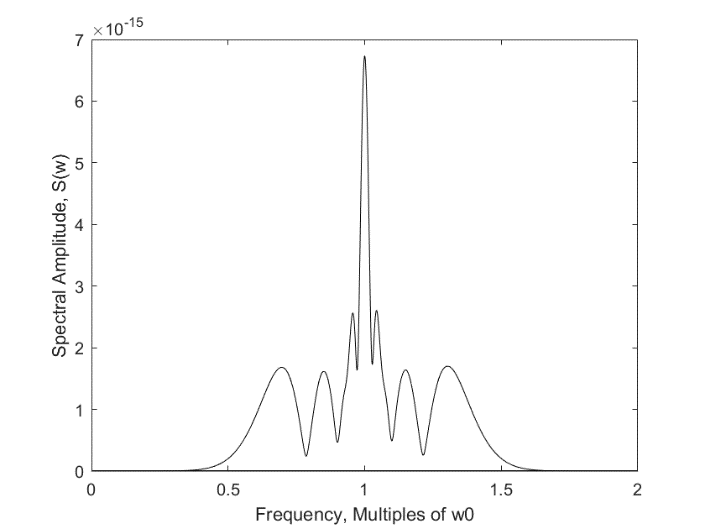  **D** |
| Reseponse time = 0.5 fs | |
| 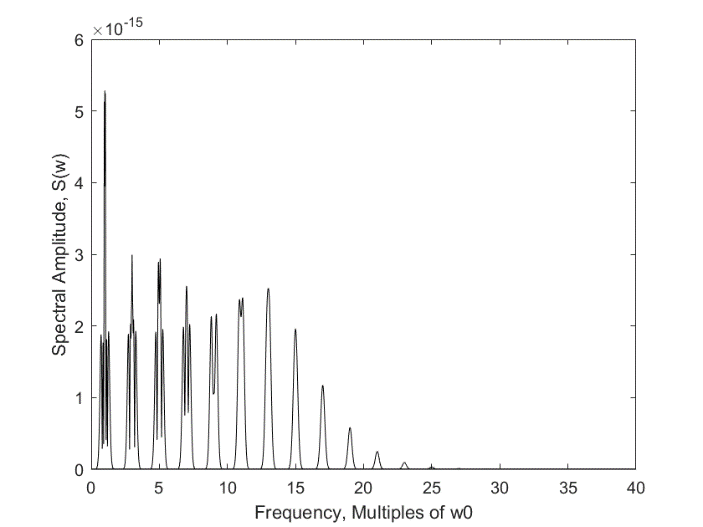  **E** | 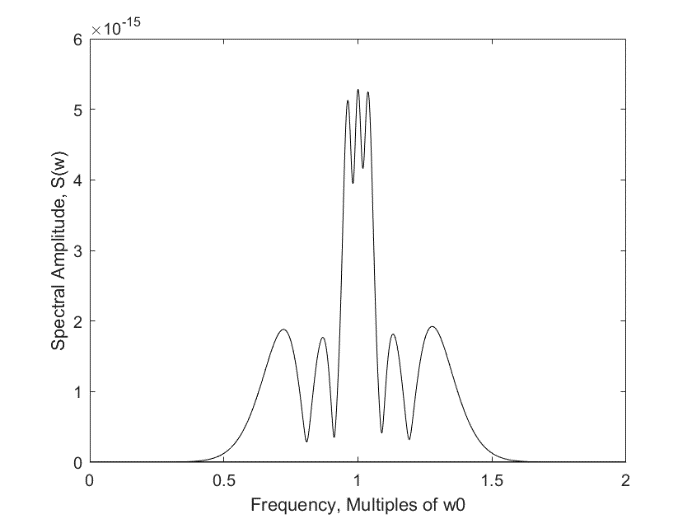  **F** |
| Reseponse time = 1 fs | |
| 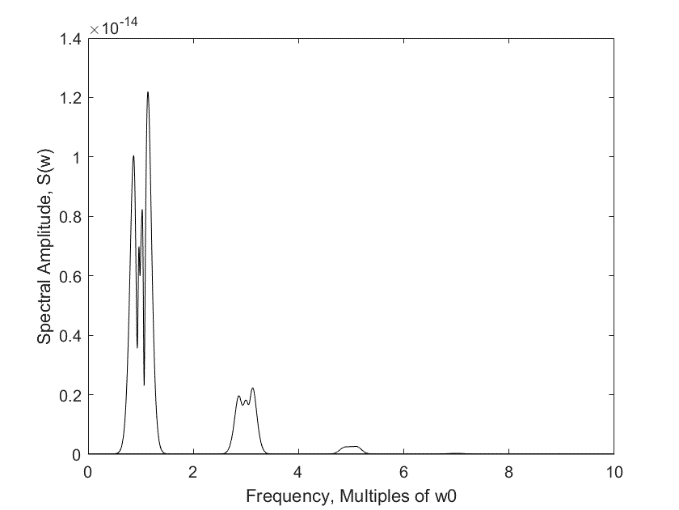  **G** | 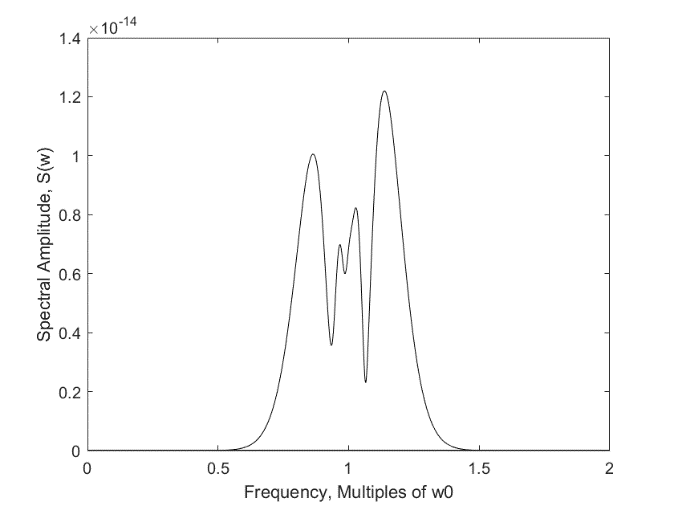  **H** |
| Reseponse time = 10 fs | |
| 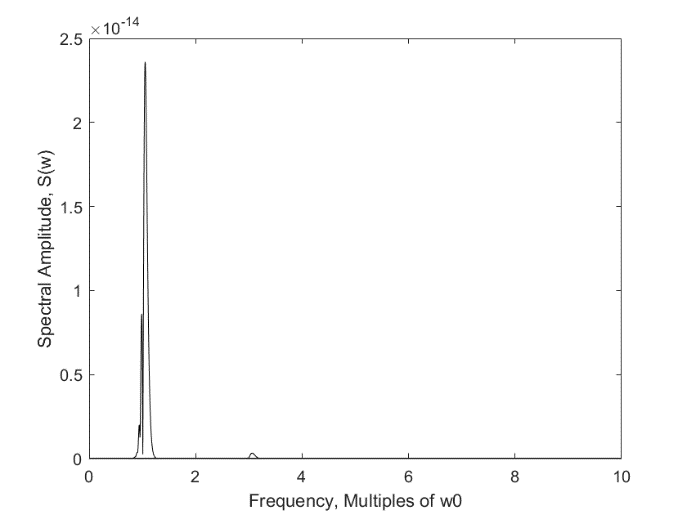  **I** | 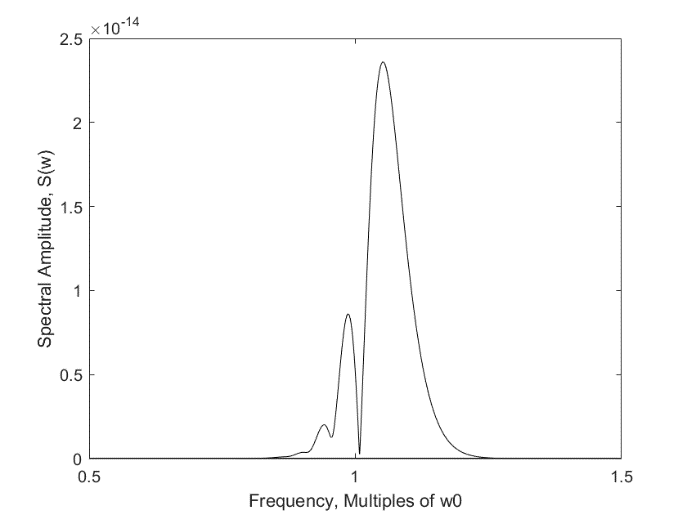  **J** |
| Reseponse time = 100 fs | |
| **Fig-S5:** HHG spectra (A,C,E,G,I) and spectral broadening of laser frequency (B,D,F,H,J) due to different response times of the propagating medium (0.1 fs for A and B; 0.5 fs for C and D; 1 fs for E and F, 10 fs for G and H, and 100 fs for I and J) for **ZnO** medium (n_0_ = 1.99 and n_2_ = 5.3x10^-15^ cm^2^/W) and the laser pulses with **wavelength = 1600 nm**, **pulse duration = 50 fs**, pulse energy = 2.63 µJ, laser spot size = 60 µm, and medium propagation distance = 0.5 mm. | |

| **HHG spectra** | **Spectral broadening of the laser pulse** |
| --- | --- |
| 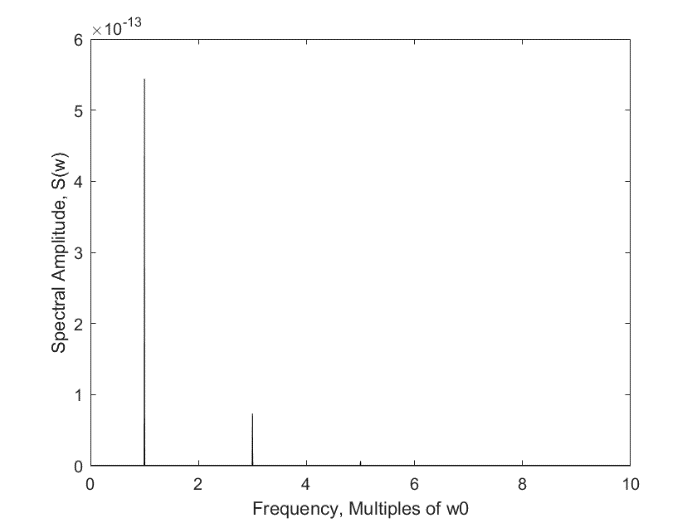  **A** | 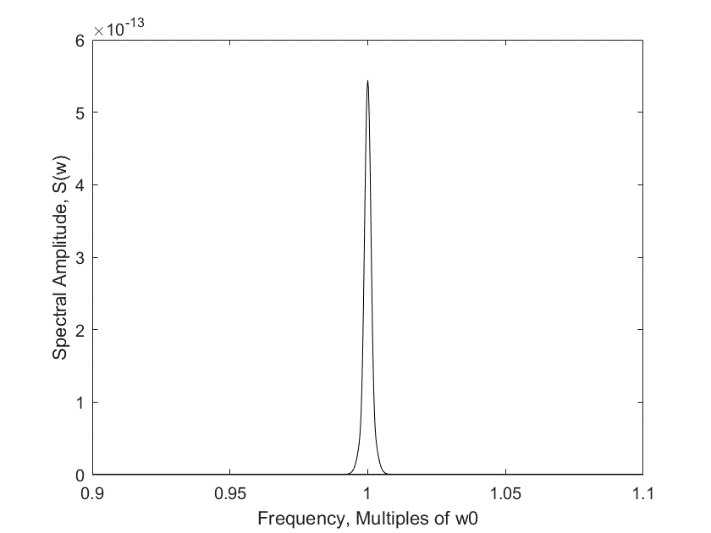  **B** |
| Reseponse time = 0.1 fs | |
| 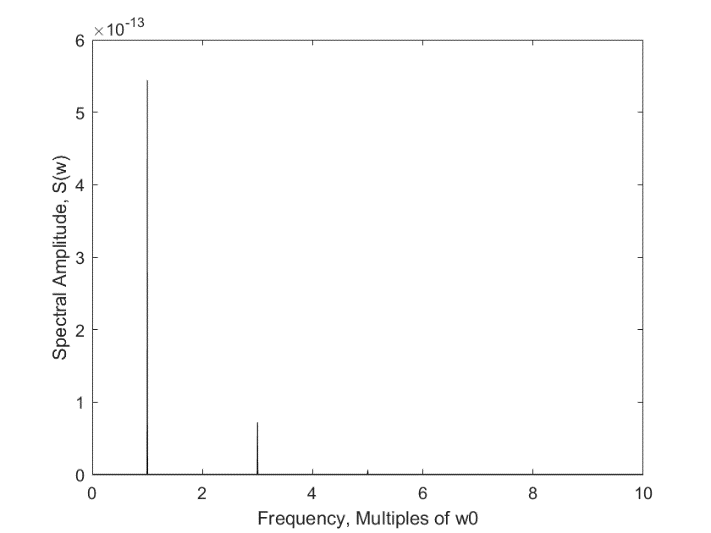  **C** | 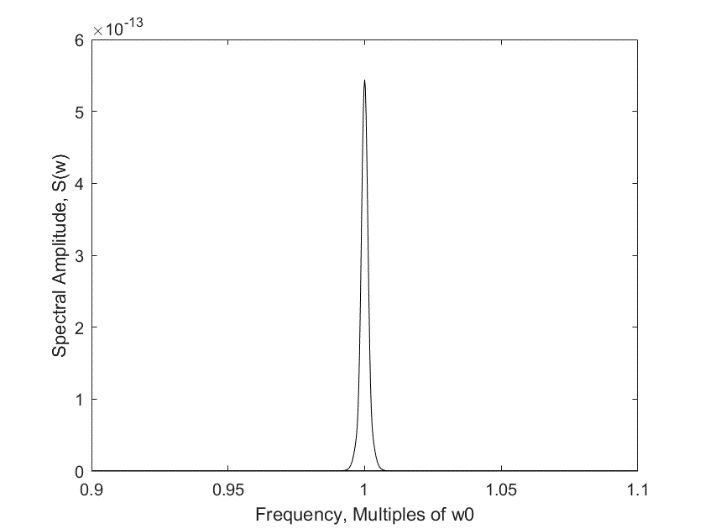  **D** |
| Reseponse time = 0.5 fs | |
| 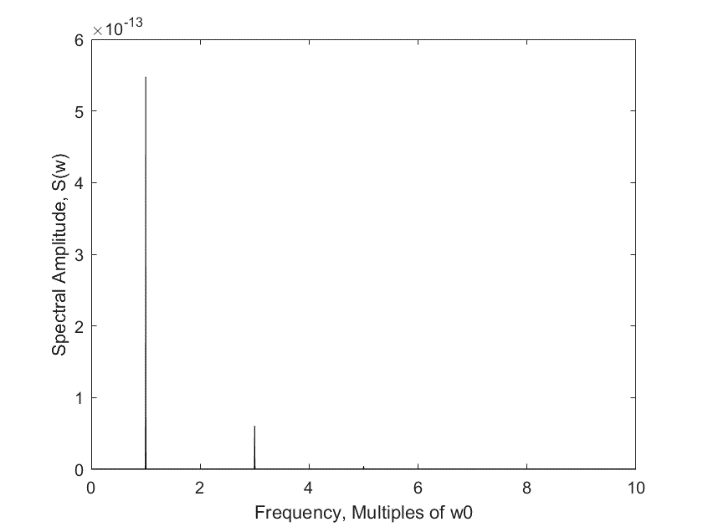  **E** | 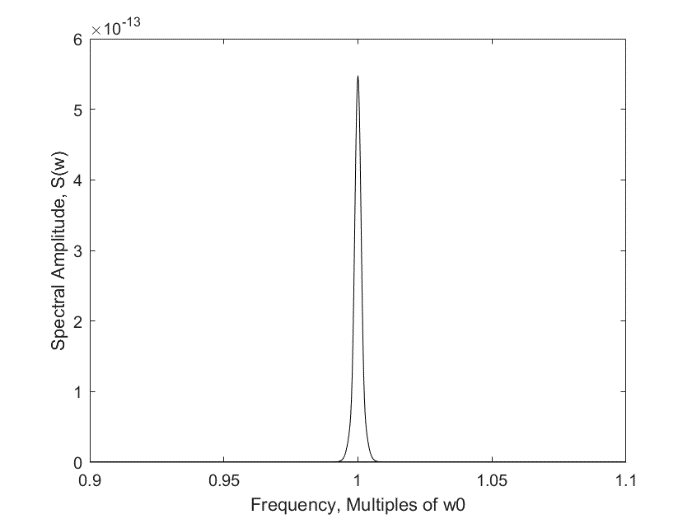  **F** |
| Reseponse time = 1 fs | |
| 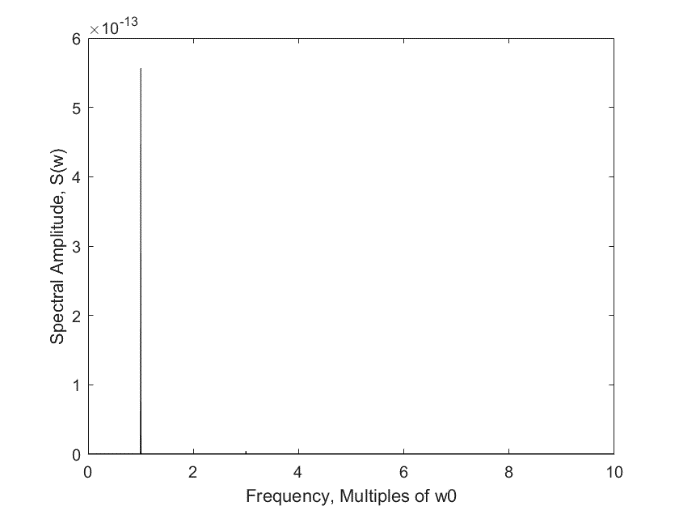  **G** | 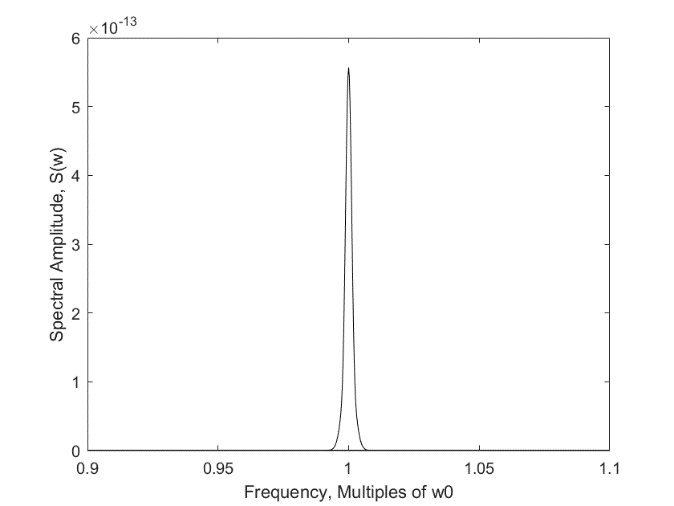  **H** |
| Reseponse time = 10 fs | |
| 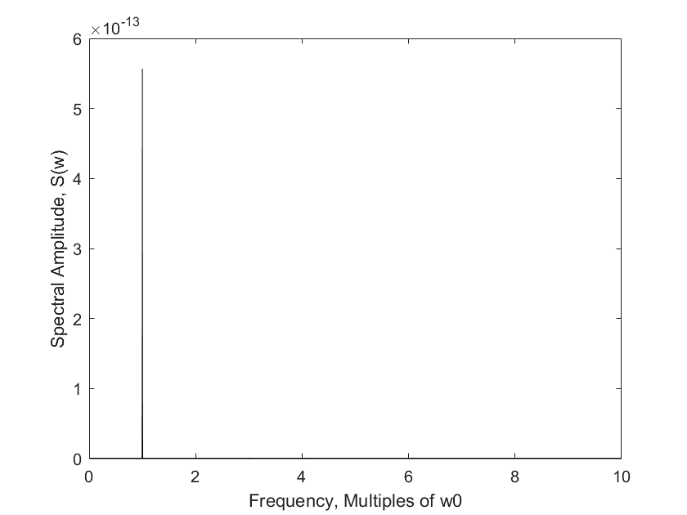  **I** | 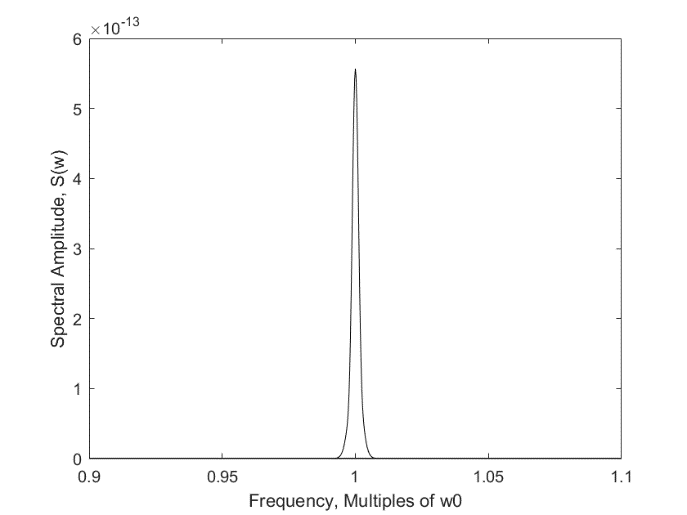  **J** |
| Reseponse time = 100 fs | |
| **Fig-S6:** HHG spectra (A,C,E,G,I) and spectral broadening of laser frequency (B,D,F,H,J) due to different response times of the propagating medium (0.1 fs for A and B; 0.5 fs for C and D; 1 fs for E and F, 10 fs for G and H, and 100 fs for I and J) for **ZnO** medium (n_0_ = 1.99 and n_2_ = 5.3x10^-15^ cm^2^/W) and the laser pulses with **wavelength = 1600 nm**, **pulse duration = 1 ps**, pulse energy = 2.63 µJ, laser spot size = 60 µm, and medium propagation distance = 0.5 mm. | |

| **HHG spectra** | **Spectral broadening of the laser pulse** |
| --- | --- |
| 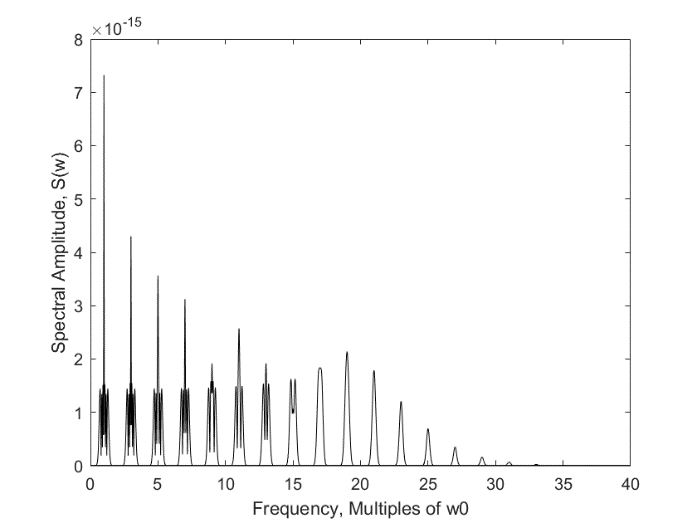  **A** | 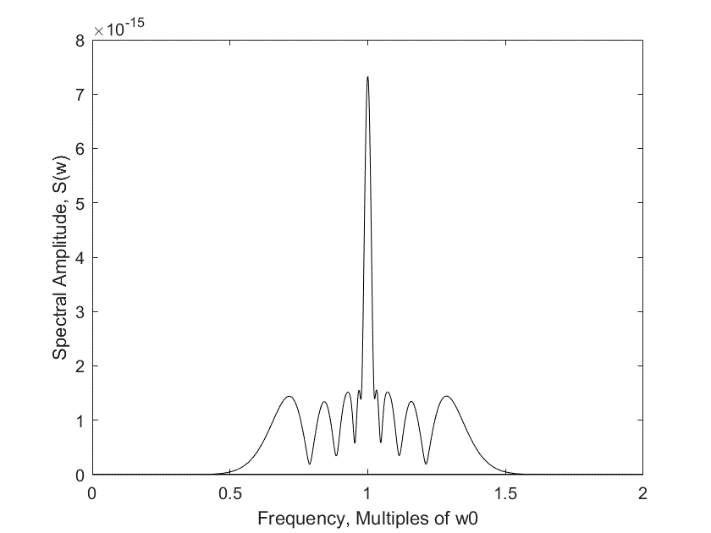  **B** |
| Reseponse time = 0.1 fs | |
| 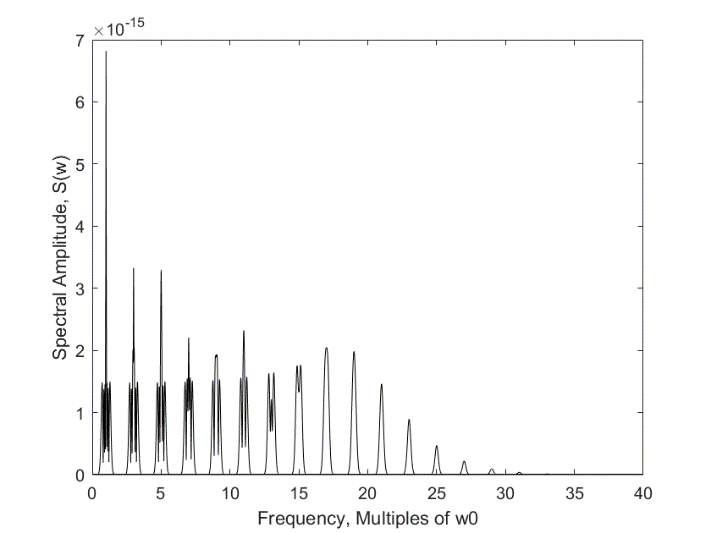  **C** | 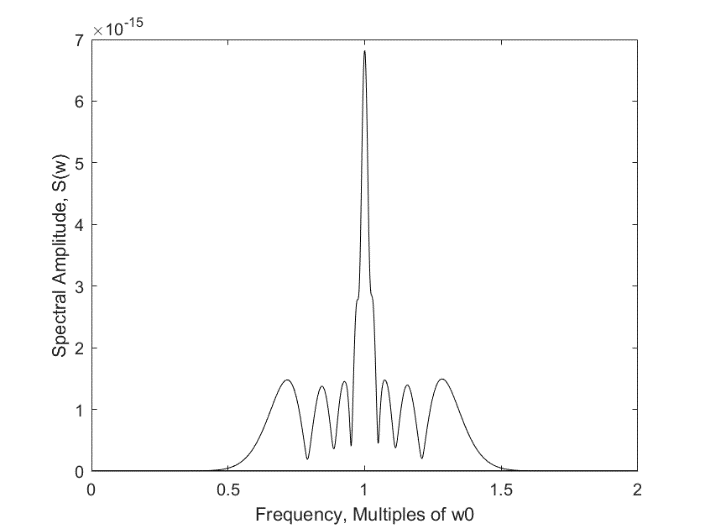  **D** |
| Reseponse time = 0.5 fs | |
| 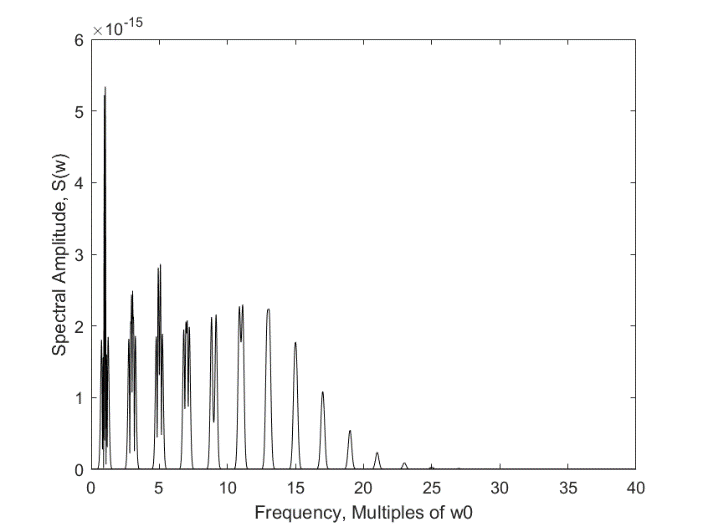  **E** | 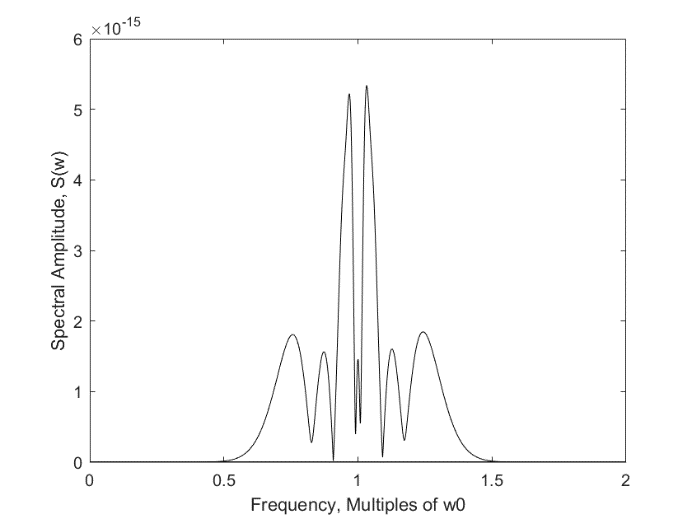  **F** |
| Reseponse time = 1 fs | |
| 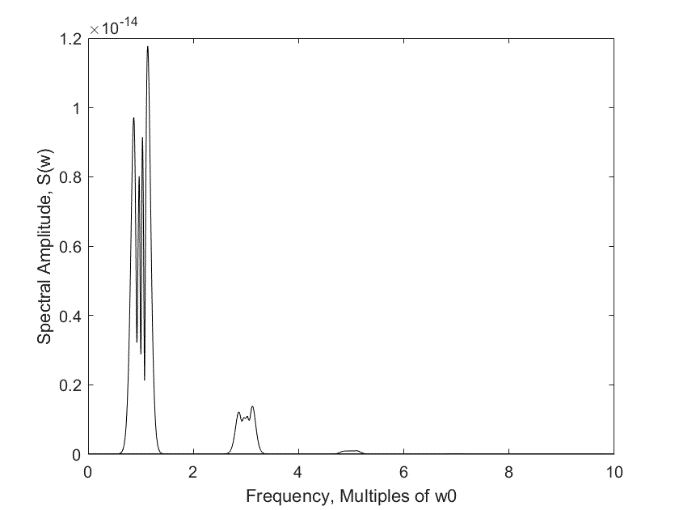  **G** | 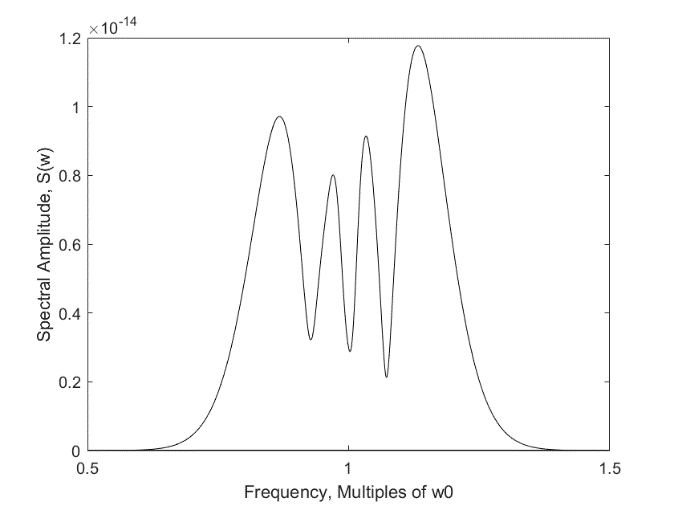  **H** |
| Reseponse time = 10 fs | |
| 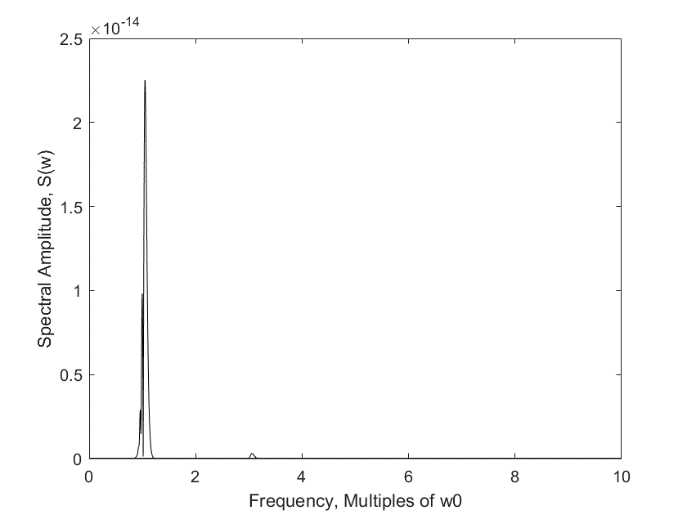  **I** | 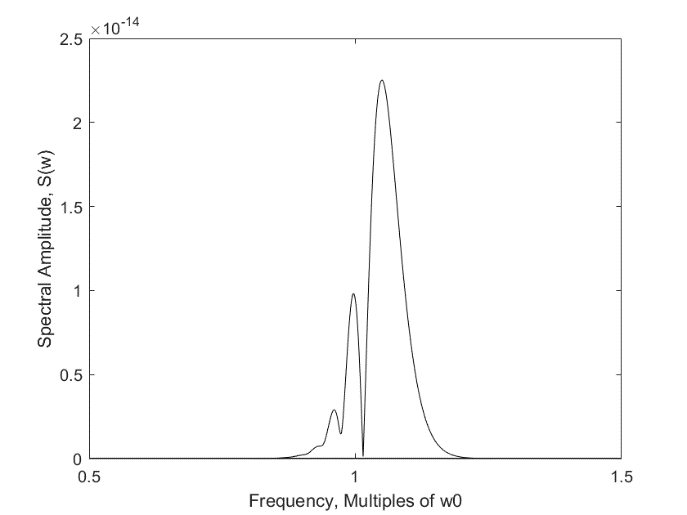  **J** |
| Reseponse time = 100 fs | |
| **Fig-S7:** HHG spectra (A,C,E,G,I) and spectral broadening of laser frequency (B,D,F,H,J) due to different response times of the propagating medium (0.1 fs for A and B; 0.5 fs for C and D; 1 fs for E and F, 10 fs for G and H, and 100 fs for I and J) for **LBG** medium (n_0_ = 1.5 and n_2_ = 4.3x10^-15^ cm^2^/W) and the laser pulses with **wavelength = 1240 nm**, **pulse duration = 50 fs**, pulse energy = 3 µJ, laser spot size = 60 µm, and medium propagation distance = 0.5 mm. | |

| **HHG spectra** | **Spectral broadening of the laser pulse** |
| --- | --- |
| 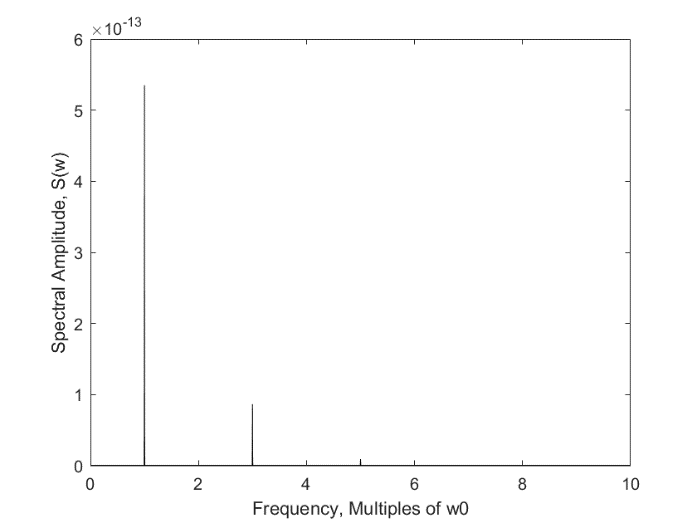  **A** | 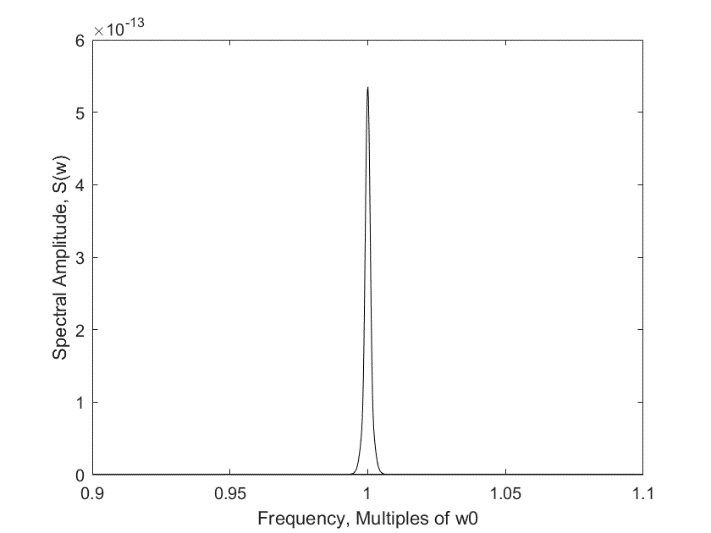  **B** |
| Reseponse time = 0.1 fs | |
| 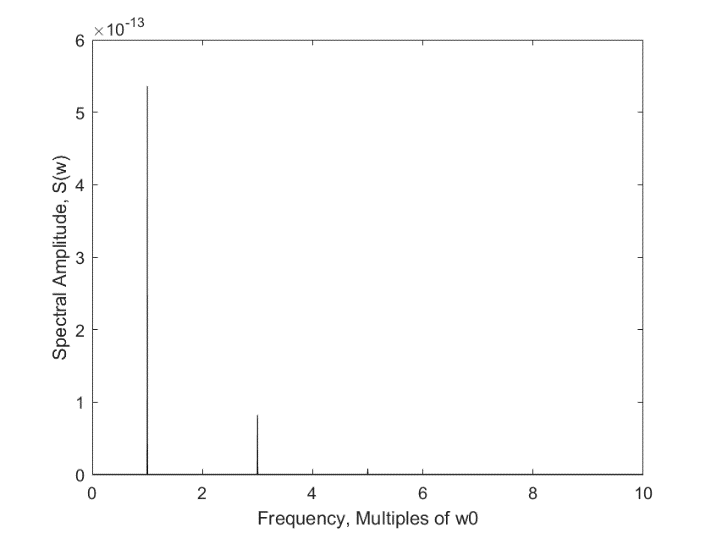  **C** | 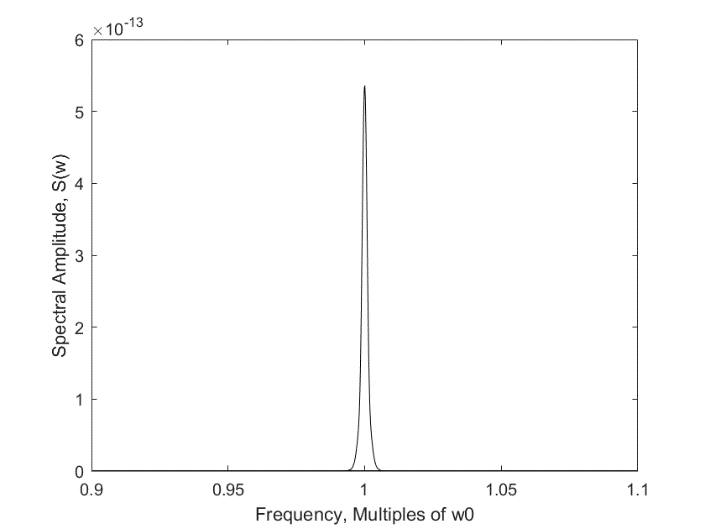  **D** |
| Reseponse time = 0.5 fs | |
| 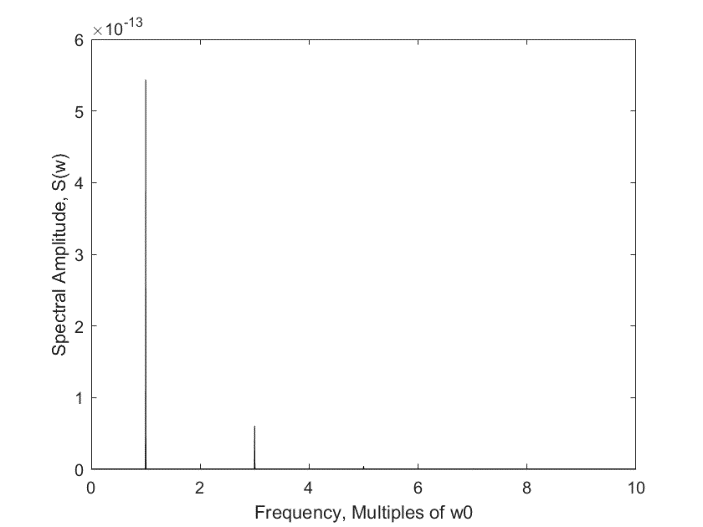  **E** | 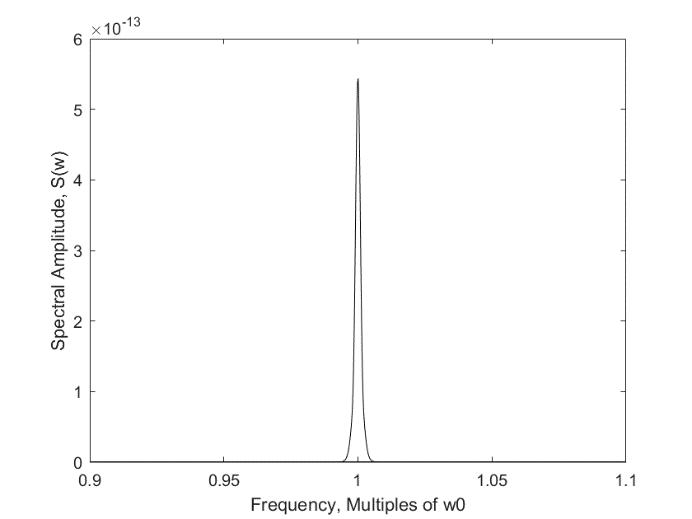  **F** |
| Reseponse time = 1 fs | |
| 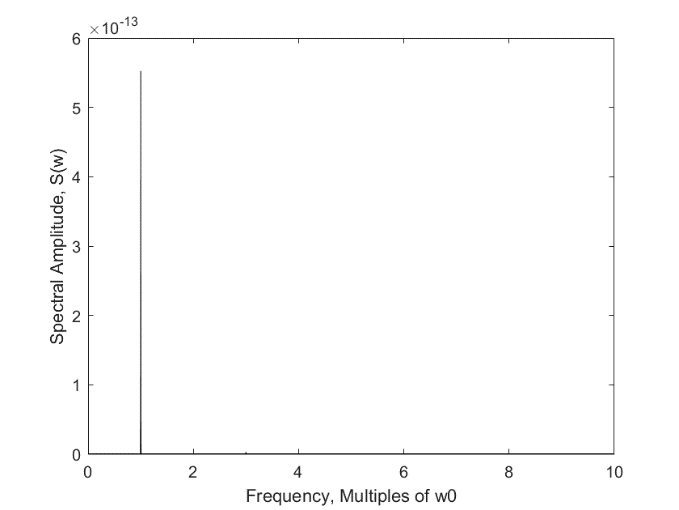  **G** | 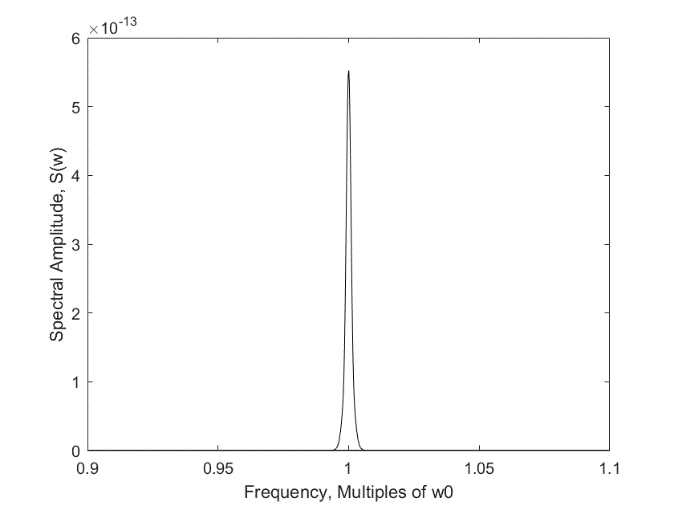  **H** |
| Reseponse time = 10 fs | |
| 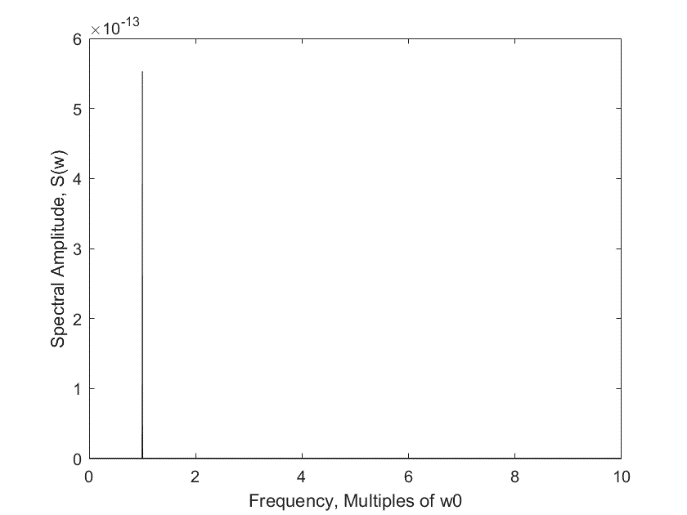  **I** | 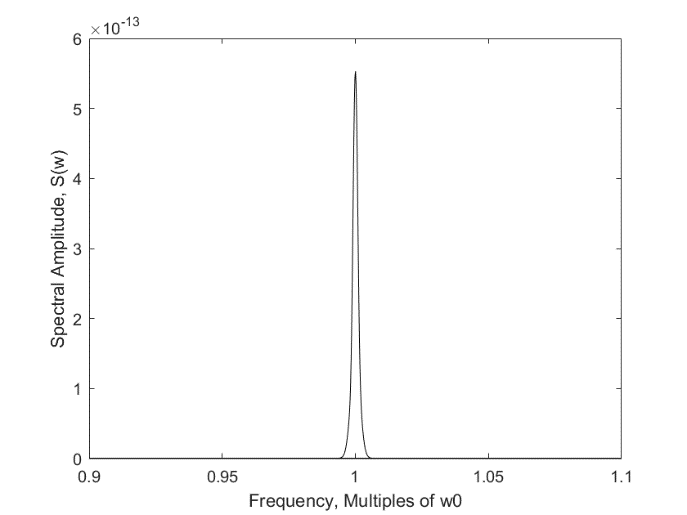  **J** |
| Reseponse time = 100 fs | |
| **Fig-S8:** HHG spectra (A,C,E,G,I) and spectral broadening of laser frequency (B,D,F,H,J) due to different response times of the propagating medium (0.1 fs for A and B; 0.5 fs for C and D; 1 fs for E and F, 10 fs for G and H, and 100 fs for I and J) for **LBG** medium (n_0_ = 1.5 and n_2_ = 4.3x10^-15^ cm^2^/W) and the laser pulses with **wavelength = 1240 nm**, **pulse duration = 1 ps**, pulse energy = 3 µJ, laser spot size = 60 µm, and medium propagation distance = 0.5 mm. | |

| 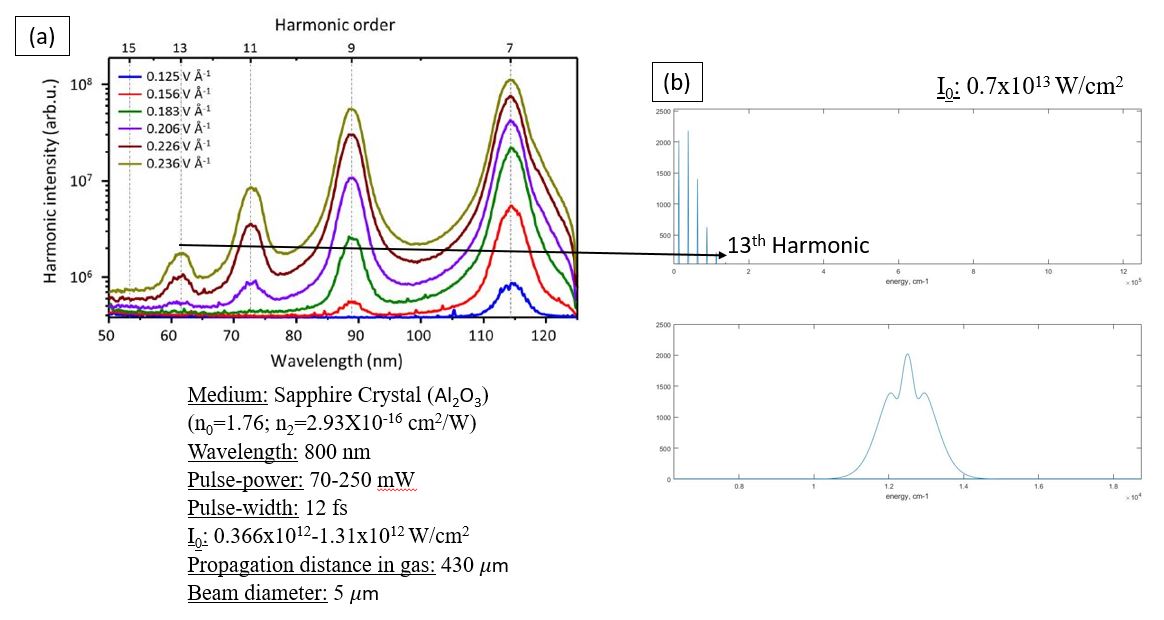 |
| --- |
| **Fig-S9:** a) Experimentally [S6] produced HHG of the sapphire (Al_2_O_3_) crystal and b) theoretical prediction from the ESPM model for the adjustable beam intensity. |

| 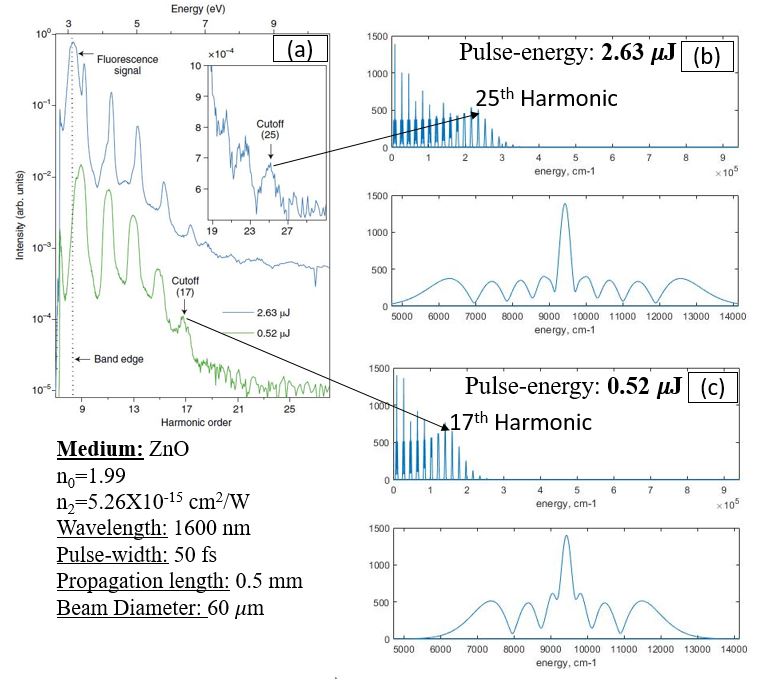 |
| --- |
| **Fig-S10:** Comparison between: a) the experimental result of HHG in ZnO [S7] and the theoretical prediction of HHG from the ESPM model in ZnO at b) 2.63 𝜇J and c) 0.52 𝜇J. This figure has been taken from our earlier work on HHG comparing experiment to the EM theory [S8] where there is an error in the figure caption using 𝜇m instead of 𝜇J. |

| 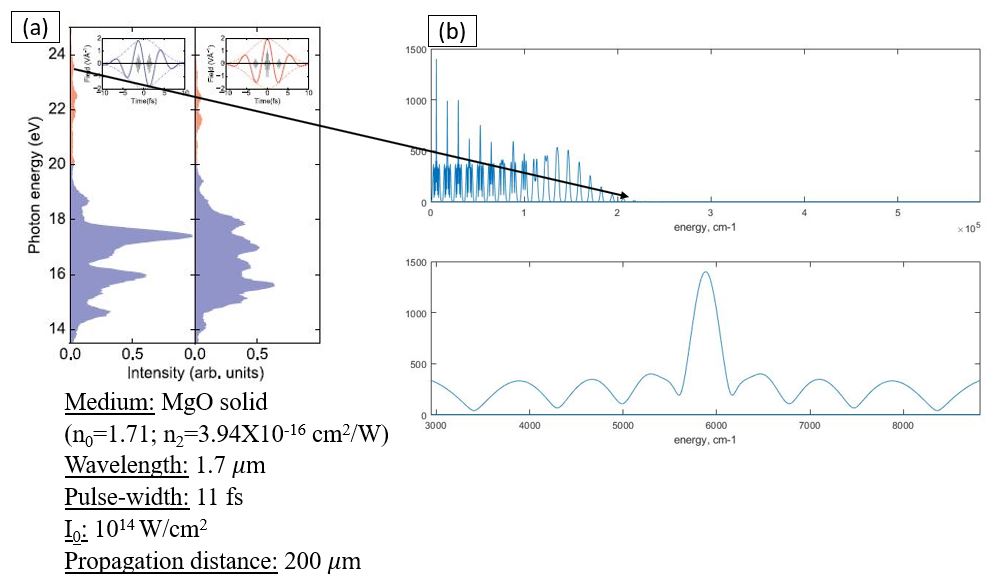 |
| --- |
| **Fig-S11:** a) Experimentally [S9] produced HHG of the MgO Solid and b) theoretical prediction from the ESPM model for the adjustable beam intensity. |

| 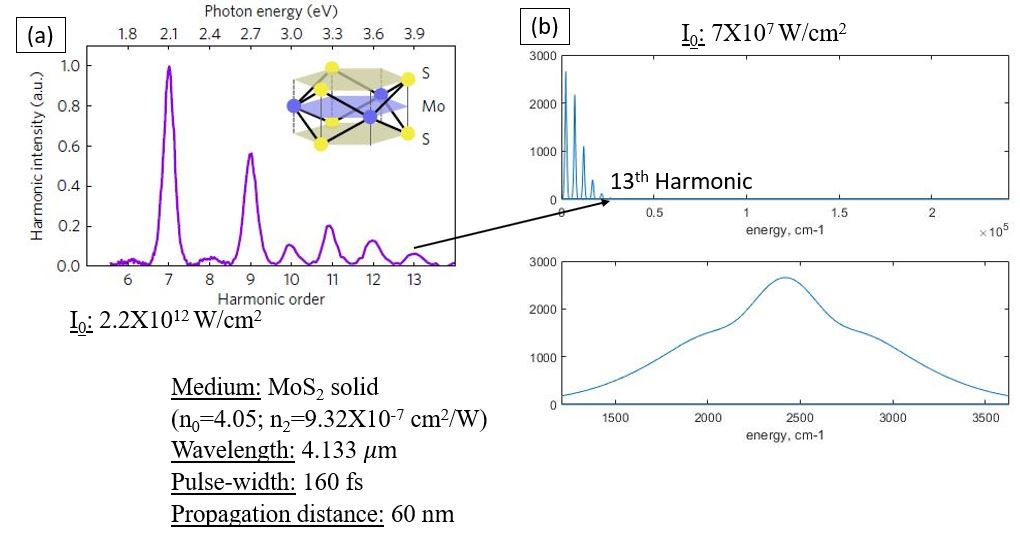 |
| --- |
| **Fig-S12:** a) Experimentally [S10] produced HHG of the MoS_2_ Solid and b) theoretical prediction from the ESPM model for the adjustable beam intensity. The generation of even harmonics in HHG demonstrates that the 3-step model is not adequate to explain even harmonics, but the EM model can easily explain the generation of even harmonics by the n_1_E term. |

**Supplementary References:**

1. R. R. Alfano, L. L. Hope, and S. L. Shapiro, “Electronic Mechanism for Production of Self-Phase Modulation”, Phys. Rev. A **6**, 433 (1972).
2. M. Lewenstein, Ph. Balcou, M. Yu. Ivanov, Anne L’Huillier, and P. B. Corkum, “Theory of high-harmonic generation by low-frequency laser fields”, Phys. Rev. A **49**, 2117 (1994).
3. R. R. Alfano and S. L. Shapiro, “Direct Distortion of Electronic Clouds of Rare-Gas Atoms in Intense Electric Fields”, Phys. Rev. Lett. **24**, 1217 (1970).
4. R. R. Alfano and S. L. Shapiro, “Observation of Self-Phase Modulation and Small-Scale Filaments in Crystals and Glasses”, Phys. Rev. Lett. **24**, 592 (1970).
5. R. R. Alfano and S. L. Shapiro, “Emission in the Region 4000 to 7000 Å Via Four-Photon Coupling in Glass”, Phys. Rev. Lett. **24**, 584 (1970).
6. H. Kim, S. Han, Y. W. Kim, S. Kim, and S. W. Kim, “Generation of Coherent Extreme-Ultraviolet Radiation from Bulk Sapphire Crystal”, ACS Photonics **4**, 1627−1632 (2017).
7. S. Ghimire, A. D. DiChaiara, E. Sistunk, P. Agostini, L. F. DiMauro, and D. A. Reis, “Observation of high-order harmonic generation in a bulk crystal”, Nat. Phys. **7**, 138-141 (2011).
8. R. R. Alfano, S. F. B. Mazhar, and L. Shi, “Higher harmonic and supercontinuum generation arising from electronic self-phase modulation under ultrafast laser pulses for various states of matter”, Optik **247**, 167872 (2021).
9. H. Kim, S. Han, Y. W. Kim, S. Kim, and S.-W. Kim, “Generation of Coherent Extreme-Ultraviolet Radiation from Bulk Sapphire Crystal”, ACS Photonics **4**, 1627−1632 (2017).
10. H. Liu, Y. Li, Y. S. You, S. Ghimire, T. F. Heinz, and D. A. Reis, “High-harmonic generation from an atomically thin semiconductor”, Nat. Phys. **13**, 262-265 (2017).
